# IL-1β Production through the NLRP3 Inflammasome by Hepatic Macrophages Links Hepatitis C Virus Infection with Liver Inflammation and Disease

**DOI:** 10.1371/journal.ppat.1003330

**Published:** 2013-04-25

**Authors:** Amina A. Negash, Hilario J. Ramos, Nanette Crochet, Daryl T. Y. Lau, Brian Doehle, Neven Papic, Don A. Delker, Juandy Jo, Antonio Bertoletti, Curt H. Hagedorn, Michael Gale

**Affiliations:** 1 Center for the Study of Hepatitis C Virus Infection and Immunity, Department of Immunology, University of Washington, Seattle, Washington, United States of America; 2 Liver Center, Division of Gastroenterology and Hepatology, Department of Medicine, Beth Israel Deaconess Medical Center, Harvard Medical School, Boston, Massachusetts, United States of America; 3 Division of Gastroenterology, Hepatology and Nutrition, Department of Medicine, University of Utah, Salt Lake City, Utah, United States of America; 4 Viral Hepatitis Laboratory, Singapore Institute for Clinical Sciences, Agency of Science Technology and Research (A*STAR), Singapore; 5 Program Emerging Viral Diseases Unit, Duke-NUS Graduate Medical School, Singapore; McMaster University, Canada

## Abstract

Chronic hepatitis C virus (HCV) infection is a leading cause of liver disease. Liver inflammation underlies infection-induced fibrosis, cirrhosis and liver cancer but the processes that promote hepatic inflammation by HCV are not defined. We provide a systems biology analysis with multiple lines of evidence to indicate that interleukin-1β (IL-1β) production by intrahepatic macrophages confers liver inflammation through HCV-induced inflammasome signaling. Chronic hepatitis C patients exhibited elevated levels of serum IL-1β compared to healthy controls. Immunohistochemical analysis of healthy control and chronic hepatitis C liver sections revealed that Kupffer cells, resident hepatic macrophages, are the primary cellular source of hepatic IL-1β during HCV infection. Accordingly, we found that both blood monocyte-derived primary human macrophages, and Kupffer cells recovered from normal donor liver, produce IL-1β after HCV exposure. Using the THP-1 macrophage cell-culture model, we found that HCV drives a rapid but transient caspase-1 activation to stimulate IL-1β secretion. HCV can enter macrophages through non-CD81 mediated phagocytic uptake that is independent of productive infection. Viral RNA triggers MyD88-mediated TLR7 signaling to induce IL-1β mRNA expression. HCV uptake concomitantly induces a potassium efflux that activates the NLRP3 inflammasome for IL-1β processing and secretion. RNA sequencing analysis comparing THP1 cells and chronic hepatitis C patient liver demonstrates that viral engagement of the NLRP3 inflammasome stimulates IL-1β production to drive proinflammatory cytokine, chemokine, and immune-regulatory gene expression networks linked with HCV disease severity. These studies identify intrahepatic IL-1β production as a central feature of liver inflammation during HCV infection. Thus, strategies to suppress NLRP3 or IL-1β activity could offer therapeutic actions to reduce hepatic inflammation and mitigate disease.

## Author Summary

Hepatitis C virus (HCV) causes chronic infection of the liver and is a leading cause of liver inflammation, cirrhosis and liver cancer in nearly 200 million people worldwide. Importantly, hepatic inflammation during chronic HCV infection is considered to be the primary catalyst for progressive liver disease and development of liver cancer. However, the underlying molecular mechanism(s) of HCV-mediated hepatic inflammation are not well understood. The goal of this study was to determine the mechanisms of HCV-induced inflammation. We found that serum IL-1β levels are elevated in chronic hepatitis C patients. Furthermore, we found that hepatic macrophages or Kupffer cells are the major IL-1β-producing cell population within HCV infected livers. Our studies, using the THP1 cell culture model of HCV exposure, reveal that exposure of macrophages to HCV induces IL-1β through a process of infection-independent phagocytic virus uptake that triggers signaling through MyD88/TLR7 and NLRP3 inflammasome pathways to drive IL-1β expression and maturation/secretion, respectively. RNA sequencing (RNA-seq) analysis of patient liver biopsies shows that viral triggering of these signaling pathways drives an inflammatory response linked with liver disease in patients with chronic hepatitis C. Our results identify HCV-induced IL-1β production by hepatic macrophages as a critical and central process that promotes liver inflammation and disease.

## Introduction

Chronic inflammation is a major contributor to disease and is the basis of hepatitis C virus (HCV)-mediated liver damage. HCV is a hepatotropic, enveloped virus that carries a single-stranded positive-sense RNA genome, and chronically infects nearly 3% of the world's population [1]. HCV productively infects hepatocytes to induce liver inflammation and progressive tissue damage leading to fibrosis and cirrhosis. These processes underlie liver dysfunction and are thought to drive the onset of liver cancer [2], [3]. However, the molecular mechanism(s) by which HCV stimulates hepatic inflammation are not defined. Interleukin-1β (IL-1β) is a central component of the cytokine milieu that accompanies both acute and chronic inflammation and viral disease [4], [5]. During microbial infection, IL-1β production is induced by cellular sensing of pathogen-associated molecular pattern (PAMP) [6], [7] motifs within microbial macromolecules and/or by metabolic products that accumulate from infection. Production of active IL-1β requires two signals, “signal one” to activate NF-κB in stimulated cells and induce IL-1β mRNA expression, and “signal two” to activate a Nod-like receptor (NLR) to promote downstream caspase-1 cleavage and processing of proIL-1β into a biologically active, secreted cytokine [8], [9]. Virus infection has been shown to induce IL-1β production through inflammasome signaling [10]. In particular, *flavivirus* agents related to HCV, including West Nile virus and Japanese encephalitis virus, trigger IL-1β production through the NLRP3 inflammasome to render immune regulation [11], [12]. Though the spectrum of IL-1β responsive genes within the liver has not been defined, IL-1β is thought to mediate its inflammatory actions by inducing the expression of proinflammatory genes, recruiting immune cells to the site of infection, and by modulating infiltrating cellular immune-effector actions [4], [13]. As a pleiotropic inflammatory factor, IL-1β has also been implicated in promoting tissue pathology and inducing the production of profibrogenic mediators [14]–[17], thereby underscoring its potential role in HCV disease.

HCV is a human blood-borne virus, of which acute exposure most often progresses to chronic infection [18] during which persistent viremia reaches levels greater than 10^6^ viral genome equivalents/cc blood in many patients [18], [19]. The unique architecture of the liver, in which blocks of hepatocytes are separated by spaces or “sinusoids” that support blood flow and dispersion of metabolites, facilitates viremia while serving to constantly expose resident hepatic cells and blood and liver-infiltrating myeloid cells to the virus. In infected hepatocytes, viral RNA recognition by retinoic acid inducible gene-I (RIG-I), the essential pathogen recognition receptor (PRR) of HCV infection [20], signals innate immune-dependent activation of transcription factors to induce type 1 interferon (IFN) and the expression of interferon-stimulated genes (ISGs) that suppress viral replication. However, HCV evades hepatocyte innate immunity through the actions of the viral NS3/4A protease that targets and cleaves mitochondrial antiviral signaling protein (MAVS) also known as IPS-1 (herein referred to as MAVS), the adaptor protein of RIG-I signaling; thus disrupting innate immunity and promoting chronic infection [21]. Moreover, exposure of hepatic stellate cells to HCV, viral antigens, or inflammatory signals can induce their fibrogenic activity to promote liver damage [22], whereas HCV exposure of hepatic myeloid cells, including resident macrophages/Kupffer cells and dendritic cells, induces cell activation to promote immune response regulation [23], [24]. In particular, Kupffer cells, the intrahepatic macrophages, play critical roles in microbial surveillance in which constant phagocytic uptake of macromolecules from hepatic sinusoidal blood provides the means for liver-wide pathogen detection. Kupffer cells account for approximately 15% of the total liver cell population [25]. Importantly, Kupffer cells can internalize HCV through a process of phagocytosis wherein internalized viral products can induce innate immune signaling of IFN-β expression [26]. This process might also induce proinflammatory cytokines that serve to recruit immune cells to the site of infection, thus supporting immune-mediated liver damage characteristic of chronic hepatitis C. Defining the mechanisms of hepatic inflammation induction by HCV is paramount for establishing attractive approaches to minimize HCV-related liver disease.

In this report we reveal a linkage of hepatic inflammation and disease in chronic hepatitis C patients attributed to IL-1β production by liver macrophages. Our observations define the hepatic macrophage/HCV interface and IL-1β production through the NLRP3 inflammasome as critical features underlying liver disease in chronic HCV infection.

## Results

### IL-1β associates with hepatic disease and is produced by liver macrophages in chronic hepatitis C patients

**Figure 1:**
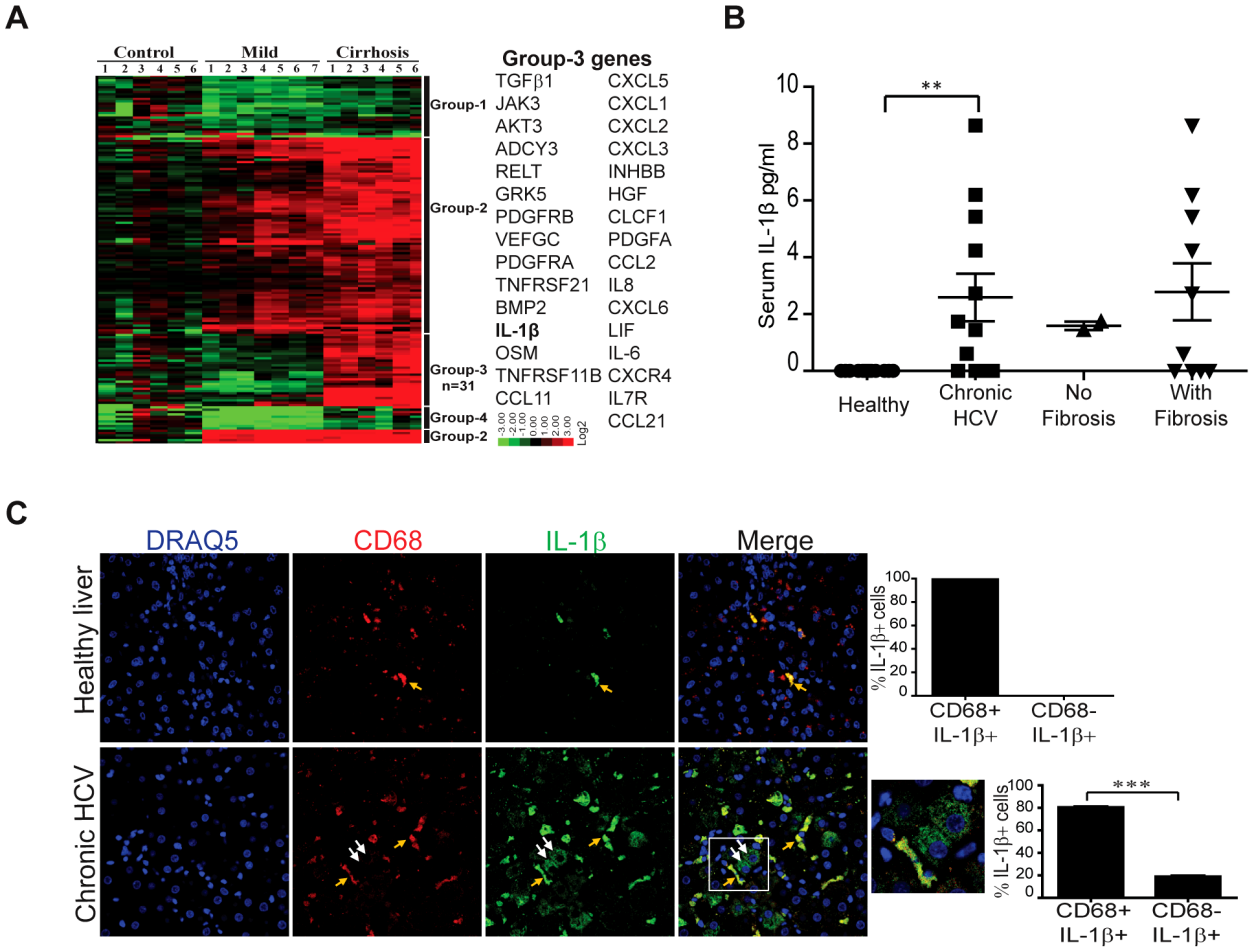
IL-1β associates with hepatic disease and is produced by liver macrophages in chronic hepatitis C patients. (**A**) Hierarchical clustering of differentially expressed genes as determined by RNA-seq analysis of liver specimens from control or HCV patients with mild (no fibrosis) and severe (cirrhosis) liver disease. Clustering analysis of a total of 158 differentially expressed genes (>1.5-fold change and FDR, 0.05) in the cytokine-cytokine receptor and chemokine signaling pathways is shown. The expression of group-3 genes were increased only in patients with severe liver disease; Group-3 genes and the expression key are shown at the right (for full description, see Table S1). For analysis see methods. (**B**) IL-1β levels from sera of chronic hepatitis C patients and healthy controls. (**C**) Immunohistochemical staining and confocal microscopy analysis of healthy liver and chronic hepatitis C patient liver samples. CD68 marks macrophages (Kupffer cells or infiltrating macrophages) (red), IL-1β (green), and DRAQ5 (blue) stains the nuclei. A quantification plot of CD68+IL-1β+ cells and CD68+IL-1β - of the total IL-1β+ cells is depicted from chronically infected (three patients,) and normal healthy liver samples. The area within the white box of the far right merged panel is enlarged and shown with cell frequency counts at right. **P = 0.0062 and ***p<0.0001 by student's t-test. Arrows (white) indicate hepatocytes adjacent to CD68+/IL-1β+ Kupffer cells (yellow arrows).

To assess hepatic gene expression patterns and host response processes associated with liver disease in chronic HCV infection, we conducted a systems biology analysis of the host response to HCV infection that included high-throughput transcriptional profiling of human liver coupled with *in vitro* modeling of the HCV/host interface. We first performed global transcriptome analysis using RNA-sequencing (RNA-seq) of RNA recovered from liver biopsies of normal control (donor) or chronic HCV-infected livers of patients staged according to mild (mild inflammation and no fibrosis) or severe (cirrhosis undergoing liver transplantation) disease. The initial comparison of transcriptome profiles from control and HCV patient liver identified four major gene expression patterns that differentially associated with liver disease (**Figure 1A** and **Table S1**). These analyses determined that cytokine/cytokine receptor interaction and chemokine signaling pathways were the most highly represented with 158 differentially expressed genes (>1.5-fold change and false discovery rate (FDR) 0.05). HCV patient liver displayed induced expression of a variety of immunomodulatory gene products known to associate with the inflammatory responses, including OSM, IL-6, IL-8, and TGF-β (**Group 3**, **Figure 1A**). Importantly, expression of IL-1β, a key proinflammatory cytokine, generally associated with HCV infection and the onset of liver disease. Moreover, assessment of a subset of genes expressed (**Group 2 and Group 3,** **Figure 1A**) in cells directly treated with IL-1β confirmed that a range of proinflammatory cytokines, chemokines, and signaling factors are directly induced by IL-1β (**Figure S1**). Thus, IL-1β and IL-1β–responsive proinflammatory cytokine expression associates with HCV infection and liver disease severity. To assess whether circulating IL-1β can be detected during chronic HCV infection, we evaluated serum levels of IL-1β from both chronic hepatitis C patients and healthy controls. IL-1β levels were significantly higher in individuals with chronic hepatitis C and revealed a possible linkage of increasing IL-1β with liver fibrosis, suggesting involvement of IL-1β with HCV disease (**Figure 1B**). We also assessed whether hepatocytes are the primary source of hepatic IL-1β during chronic HCV infection. Surprisingly, we found that upon either HCV exposure or infection neither immortalized primary human hepatocytes (PH5CH8 and IHH cells) nor hepatoma cells (Huh7) expressed inducible IL-1β mRNA or secreted appreciable levels of mature IL-1β (**Figure S2A, S2B**). To identify the cellular source of hepatic IL-1β during chronic HCV infection, we conducted confocal microscopy analysis of immunostained liver sections from normal donor liver or patients with chronic hepatitis C. Liver sections were co-stained with anti- IL-1β and anti-CD68, a surface marker present on macrophages such as the liver-resident myeloid Kupffer cells. We found that while CD68-negative cell populations, including hepatocytes, contained little or no IL-1β, liver macrophages from chronic hepatitis C patients expressed IL-1β, and CD68-positive cells were present at increased frequency compared with healthy controls **(** **Figure 1C** and **Figure S3**). Thus, IL-1β associates with chronic HCV infection, and our observations identify Kupffer cells and/or infiltrating liver macrophages but not hepatocytes as a primary hepatic source of IL-1β.

### HCV stimulates IL-1β production upon uptake by macrophages

**Figure 2:**
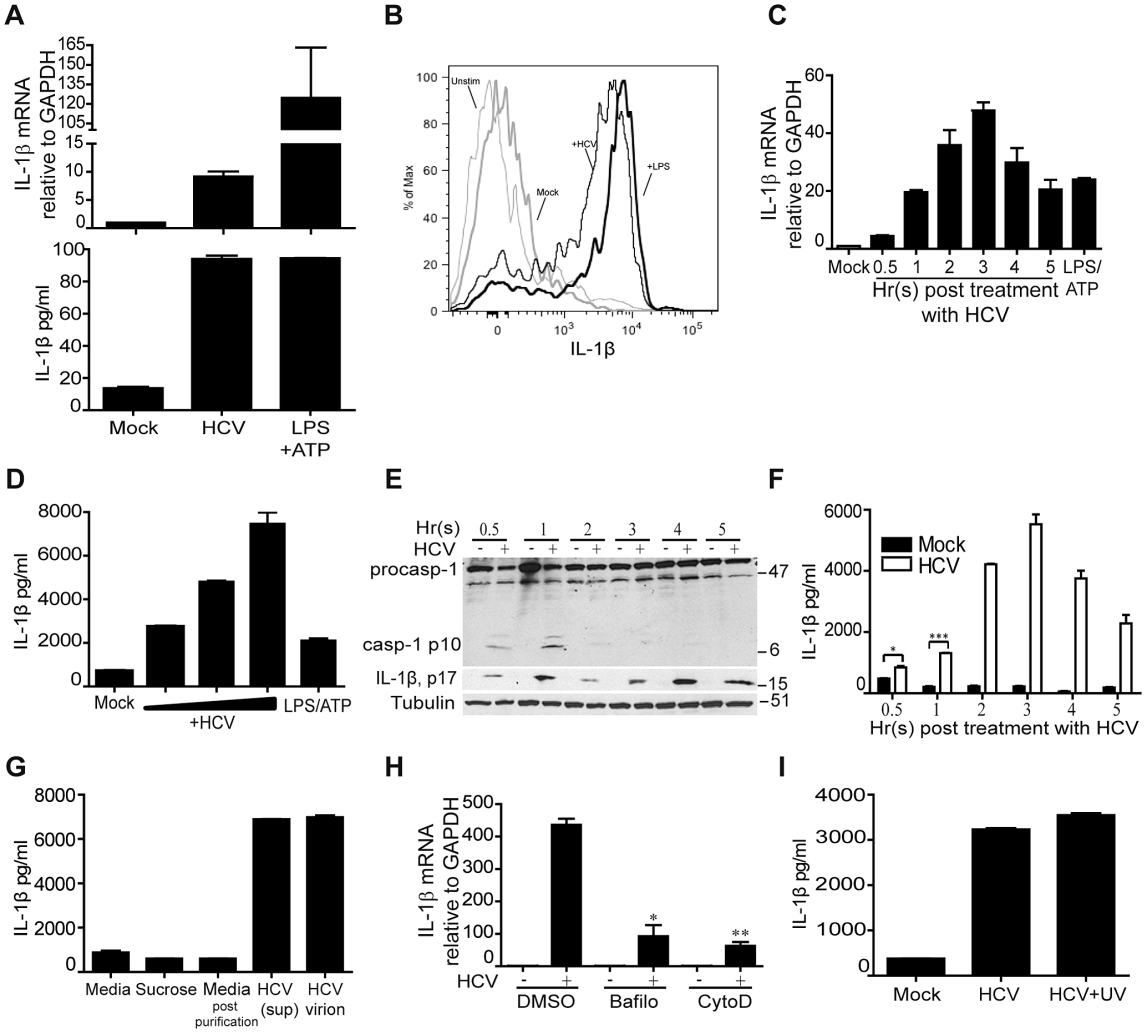
HCV stimulates IL-1β production upon uptake by macrophages. (**A**) IL-1β mRNA expression (upper panel) and secreted IL-1β protein levels (lower panel) from primary monocyte-derived macrophages of healthy human donor blood. Cells were mock-treated or treated with infectious HCV supernatant (moi = 0.01 based on Huh7 focus forming units (ffu) or treated with 1 µg/ml of LPS and ATP 5 mM (LPS/ATP; positive control). (**B**) Intracellular cytokine staining of treated CD14+ cells recovered from saline washout of healthy donor liver. Cells were left untreated (unstim) or were cultured with conditioned media (mock, negative control), LPS (positive control) or treated with UV-inactivated HCV (moi = 0.01 based on Huh7 focus forming units (ffu)). Data are shown from one donor and are representative two experiments each of cells collected from two independent donors. In the analysis shown the frequency of IL-1β-expressing cells was: unstim, 2.7%; mock, 6.4%; LPS, 76.5%; UV-HCV. 67.6%. (**C**)–(**I**) Analysis of THP1 cells. (**C**) IL-1β mRNA expression post exposure to HCV. (**D**) IL-1β protein secretion after treatment with variable doses of HCV (moi = 0.001, 0.01 or 0.1 Huh7 ffu) or LPS/ATP at 1 µg/ml for 24 hr. (**E**) Immunoblot showing the kinetics of caspase-1 activation after HCV exposure. (**F**) Levels of secreted IL-1β over a time course after HCV exposure (moi = 0.01 based on Huh7 ffu). (**G**) IL-1β levels secreted 24 hr after exposure to (left to right) cell culture media, sucrose solution, sucrose-purified culture media, infectious HCV supernatant or sucrose-purified HCV virions. (**H**) IL-1β mRNA expression in pre-treated with DMSO (control), bafilomycin (2.5 uM) or cytochalasin D (10 µM) cells and exposed to media or infectious HCV supernatant (moi = 0.01 Huh7 ffu). (**I**) Levels of secreted IL-1β 24 hr post treatment with conditioned media alone (mock) or treatment with live infectious HCV (HCV, moi = 0.01) or UV-inactivated HCV (HCV-UV). *P = 0.0175 and ***P = 0.0005, by student *t-test*.

To assess the contribution of hepatic macrophages to the production of IL-1β from HCV exposure, we first modeled HCV exposure of macrophages in culture using primary human blood monocyte-derived macrophages. Primary macrophages express IL-1β mRNA and secrete IL-1β upon exposure to HCV *ex vivo* (**Figure 2A**). Neither Kupffer cells nor macrophages in general are known to be productively infected with HCV [27], though in the liver they are constantly exposed to the virus through liver sinusoidal blood flow wherein exposure to HCV might trigger their production of IL-1β. To test this possibility, we collected mononuclear cells from saline wash-out of normal donor liver and treated the cells with conditioned cell culture media (mock) or media containing UV-inactivated HCV. Cells were then stained with specific antibodies to detect intracellular IL-1β and monocyte/macrophage cell surface markers (CD14 and CD68). We found that all CD68+ cells recovered from donor liver also expressed CD14, thus defining them as hepatic macrophages (data not shown). Flow cytometric analysis of these cells demonstrated that HCV exposure induced IL-1β production (**Figure 2B** **and Figure S4A, S4B**). As UV-inactivated HCV does not replicate, these observations suggest that hepatic macrophages can produce IL-1β after HCV exposure in a manner independent of direct cell infection. We therefore sought to determine how HCV exposure can drive IL-1β production from macrophages. Using an *in vitro* model of macrophage exposure to HCV based on differentiated human THP-1 cells, we found that as in primary macrophages, exposure of THP-1 cells to HCV stimulates the expression of IL-1β mRNA that peaks 3 hr after virus exposure (**Figure 2C**), leading to secretion of mature IL-1β (**Figure 2D**). THP-1 cell exposure to HCV triggers a rapid, transient activation and cleavage of caspase-1, which coincides with the production (**Figure 2E**) and secretion (**Figure 2F**) of mature IL-1β. Neither the production nor secretion of IL-1β were observed in parallel cultures of resting cells not exposed to HCV (data not shown). To address the possibility that contaminants from the HCV preparation might trigger IL-1β production and secretion, we purified HCV virions through a sucrose cushion prior to cell treatment. Both HCV-containing media and the resulting purified virions were infectious in Huh7 cells (**Figure S5**). We found that purified HCV virions stimulate IL-1β secretion in a manner comparable to IL-1β induction triggered by treatment of cells with HCV-containing supernatants (**Figure 2G**), thus demonstrating the HCV virion-specific activity of IL-1β production in macrophages.

As myeloid cells do not support HCV replication [27], we investigated how HCV virions might trigger IL-1β expression. HCV binds and enters hepatocytes through engagement of cell surface CD81, followed by association with additional co-receptor molecules [28]. However, we found that following virus exposure, HCV RNA is present in THP1 cells through a process independent of the CD81 co-receptor, demonstrating that the virus is not entering THP-1 cells via an active entry mechanism (**Figure S6A**). We therefore evaluated the role of phagocytosis in THP1 cell uptake of HCV by examining virus uptake after cell treatment with cytochalasin D (an inhibitor of phagocytosis) or the vacuolar type H+-ATPase inhibitor bafilomycin (which prevents endosome acidification). Exposure of THP-1 cells to HCV in the presence of cytochalasin D but not bafilomycin suppressed virus uptake as measured by intracellular accumulation of HCV RNA (**Figure S6B**), while treatment of cells with either inhibitor caused a significant reduction in HCV-induced IL-1β mRNA expression (**Figure 2H**). We also confirmed in the THP-1 model that the IL-1β-stimulatory activity of HCV did not require viral replication, as UV-inactivated virus induced robust IL-1β production from treated THP-1 cells as effectively as infectious HCV (**Figure 2I**). Thus, both infection-independent phagocytic-mediated uptake of HCV virions as well as endosomal acidification are involved in IL-1β stimulation in macrophages.

### HCV stimulates IL-1β expression through MyD88-dependent viral RNA signaling by Toll-like receptor (TLR) 7

**Figure 3:**
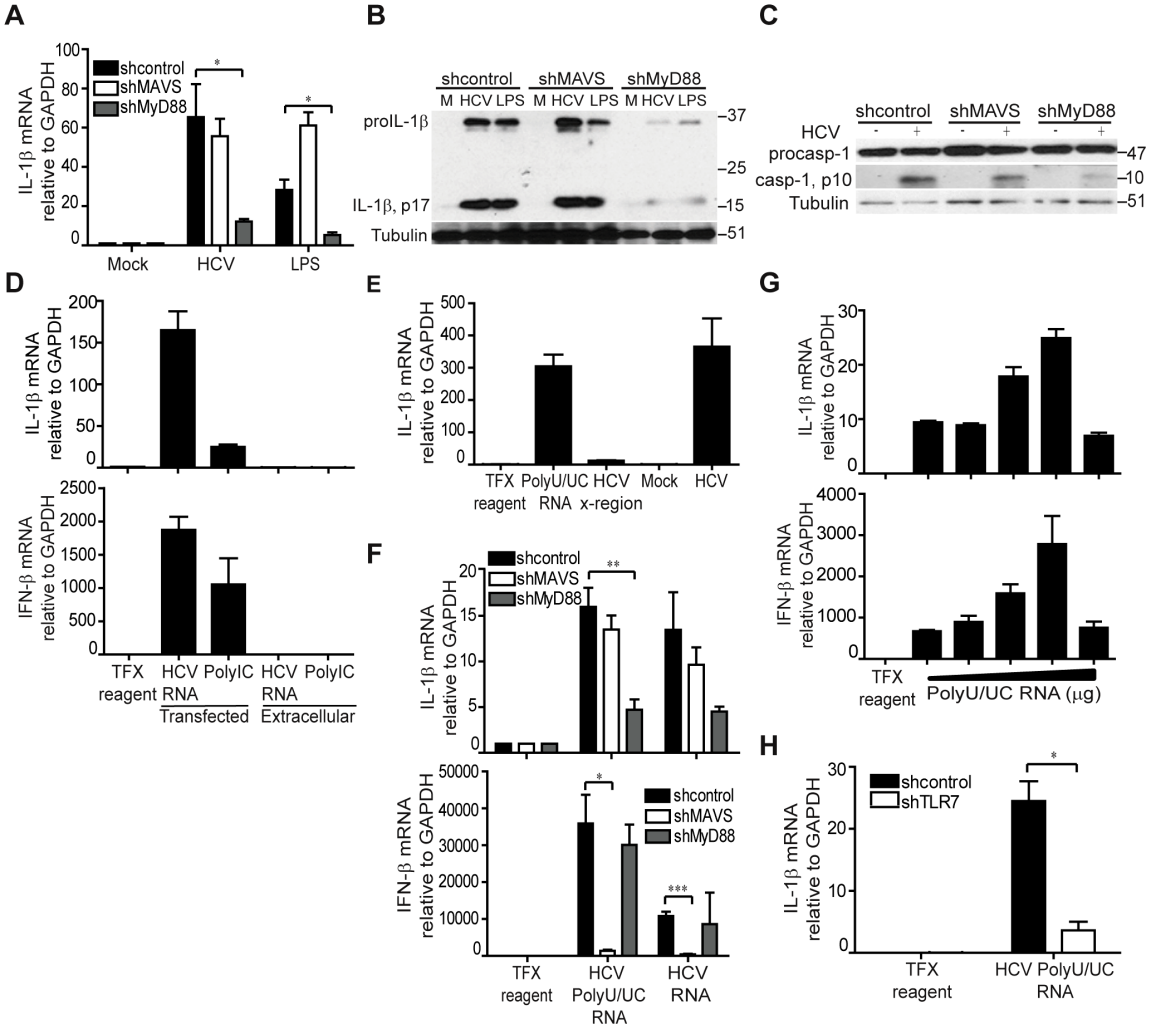
HCV stimulation of IL-1β expression occurs through MyD88-dependent viral RNA signaling by TLR7. (**A**) THP1 cells stably expressing non-targeting shRNA (control) or shRNA specific to MAVS or MyD88 were mock treated with media alone, exposed to HCV (moi = 0.01) or treated with LPS (1 µg/ml) and IL-1β expression assessed by qRT-PCR, *P = 0.0341and *P = 0.013. (**B**) Immunoblot analysis of IL-1β in THP-1 stably expressing the indicated shRNA. Cells were exposed to media alone (M) or infectious HCV supernatant for 6 hr. (**C**) Immunoblot of caspase-1 in THP-1 stably expressing the indicated shRNA. (**D**) IL-1β (upper panel) and IFN-β (lower panel) mRNA expression in THP-1 post-treatment with transfection reagent alone (control) or transfected with full length HCV RNA or poly IC (transfected); or treated with media containing full length HCV RNA or poly IC (extracellular). (**E**) IL-1β mRNA levels in THP-1 transfected with HCV polyU/UC RNA (1 µg/ml), HCV X-region (1 µg/ml) or exposed to infectious HCV for 6 hrs. (**F**) IL-1β (upper panel) and IFN-β (lower panel) mRNA expression in THP1 harboring the indicated shRNA, *P = 0. 0115, **P = 0.0094 and ***P = 0.0009. (**G**) IL-1β (upper panel) and IFN-β (lower panel) mRNA expression in THP-1 cells transfected with increasing doses (0.125, 0.25, 0.5, 1 and 2 µg/ml) of HCV polyU/UC RNA. (**H**) IL-1β mRNA expression in THP1 cells stably expressing non-targeting or shRNA specific to TLR7, *P = 0.022, by student's t-test.

To determine how HCV triggers IL-1β production in macrophages, we assessed the requirement for known PRRs in the activation of IL-1β expression. We first examined the requirement for MAVS or myeloid differentiation primary response gene 88 (MyD88)-dependent innate immune signaling in IL-1β expression. Whereas stable knockdown of MyD88 abrogated HCV-induced IL-1β mRNA induction and proIL-1β production, knockdown of MAVS had no effect on IL-1β mRNA expression (**Figure 3A, B** and **Figure S7A, S7B**). In contrast to a role for MyD88 in activation of signal one of inflammasome activation and IL-1β production, neither MyD88 nor MAVS were required for signal two that drives caspase-1 activation for processing IL-1β into its active, secreted form (**Figure 3C**). We next sought to identify the HCV component(s) that trigger IL-1β expression. Previous studies have shown that macrophage exposure to viral products can trigger IL-1β production [29] and that HCV RNA itself is a potent PAMP that triggers host innate immune response programs [20], [30]. We therefore evaluated the ability of the HCV RNA to trigger IL-1β expression in THP1 cells. We found that transfected but not extracellular HCV RNA is sufficient to stimulate both IFN-β and IL-1β mRNA expression (**Figure 3D**). By comparison, transfected synthetic double-stranded RNA, poly inosine-cytosine (polyIC), also triggers strong IFN-β mRNA expression. However, despite comparable levels of IFN induced by both polyIC and HCV, HCV RNA was a much greater stimulant of IL-1β, thus demonstrating that HCV RNA is a highly potent and specific agonist of IL-1β mRNA induction.

To define the HCV viral-RNA moiety and host PRR that drives IL-1β mRNA induction, we stimulated cells with either the HCV RNA polyU/UC region (PAMP) or the non-stimulatory X-region of the viral RNA. The HCV RNA polyU/UC region is a uridine-rich motif within the 3′ nontranslated region of HCV genome that exhibits potent activation of type I IFN and has thus been defined as a viral PAMP for RIG-I activation [30], [31]. In contrast, the HCV RNA X-region is located in the 3′ nontranslated region of the viral genome and lacks the ability to stimulate type I IFN [30]. We found that the polyU/UC but not the X-region RNA motif was sufficient to trigger IL-1β mRNA expression when introduced into THP1 cells (**Figure 3E**). Remarkably, IL-1β and IFN-β mRNA are induced through MyD88 or MAVS-dependent signaling, respectively, in response to HCV RNA in macrophages (**Figure 3F**), with both exhibiting dose dependence of polyU/UC motif concentration (**Figure 3G**, and see **Figure 3A**). Thus, the polyU/UC PAMP motif within the HCV RNA is a non-self ligand of PRR signaling through which MyD88-dependent signals impart IL-1β mRNA expression while RIG-I/MAVS signaling drives IFN-β expression. We also assessed how MyD88 induction of IL-1β is propagated in the context of HCV stimulation. Notably, TLR7 resides within endosomes and can recognize uridine-rich, single-stranded RNA as a PAMP to signal through MyD88 [32], [33]. Moreover, TLR7 is abundantly expressed in tissue macrophages and differentiated THP1 cells [34]. As bafilomycin treatment abrogates IL-1β expression induced by HCV (see **Figure 2G**), signaling from endosomal TLRs is likely important for inflammasome triggering. We therefore examined the requirement for TLR7 in HCV RNA-induced IL-1β mRNA expression. Knockdown of TLR7 expression significantly reduced signaling of IL-1β mRNA expression triggered by cell exposure to HCV (**Figure S8A** and **S8B**) or induced by HCV RNA (**Figure 3H**). Thus, “signal one” of inflammasome activation is mediated by TLR7 recognition of HCV RNA within endosomes to induce IL-1β mRNA expression through MyD88-dependent signaling upon macrophage exposure to HCV.

### The NLRP3 inflammasome and potassium efflux are required for HCV-driven maturation of IL-1β in macrophages

**Figure 4:**
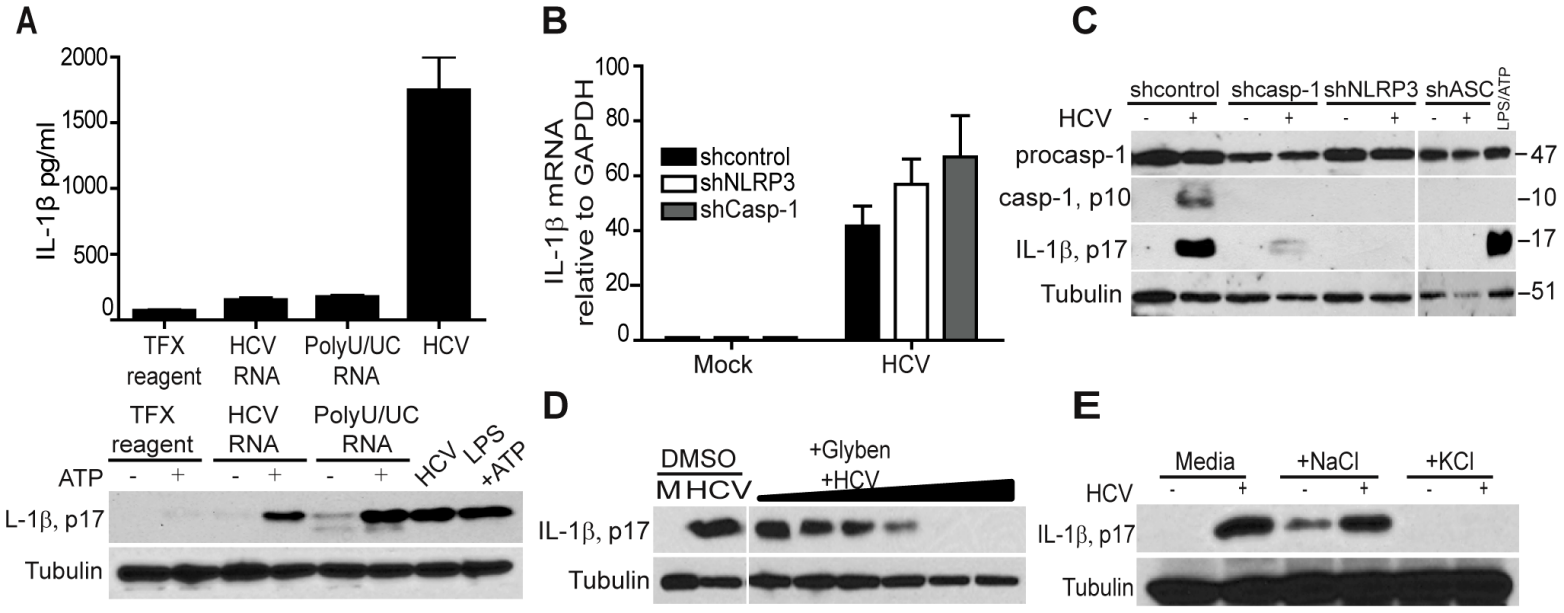
HCV triggers the NLRP3 inflammasome and IL-1β maturation through induction of potassium efflux after macrophage uptake. (**A**) Secreted IL-β protein levels (upper panel) and immunoblot analysis of IL-1β (lower panel set) of THP-1 treated with transfection reagent or transfected with either with full length HCV RNA or polyU/UC RNA or exposed to HCV (moi = 0.01). (**B**) IL-β mRNA expression in THP1 stably expressing non-targeting control shRNA or shRNA specific to NLRP3 or caspase-1. (**C**) Immunoblot of caspase-1 and IL-1β in THP1 stably expressing non-targeting control the indicated shRNA. (**D**) THP-1 were pre-treated with DMSO (control) or with 6.25, 12.5, 25, 50, 100, 200 µM of potassium channel inhibitor glybenclamide (Glyben) for 2 hrs followed by mock treatment (M; control) or HCV (moi = 0.01) exposure in the presence of glyben for an additional 1 hr. (E) IL-1β p17 abundance in THP-1 cultured in normal media or in media containing NaCl (100 mM) or KCl (100 mM) for 1 hr followed by mock-treatment (-) or exposure to HCV (moi = 0.01) in the same media for an additional 1 hr.

Comparison of proIL-1β processing in cells stimulated with HCV RNA or HCV itself demonstrated that secretion of IL-1β requires events triggered upon uptake of intact virus (**Figure 4A**), indicating that HCV must also induce the “signal two” that mediates full activation of the inflammasome leading to IL-1β secretion. To define the inflammasome responsible for IL-1β processing triggered by HCV, we examined the requirement for specific NLR signaling in IL-1β secretion. In particular, the NLRP3 inflammasome has been shown to respond to diverse stimuli, including virus infection [10], and drives proIL-1β processing during *flavivirus* infection [11], [12]. We therefore assessed IL-1β production in THP-1 cells stably expressing non-targeting shRNA or shRNA targeting caspase-1 or NLRP3. IL-1β mRNA expression was efficiently induced by HCV regardless of caspase-1 or NLRP3 knockdown (**Figure 4B** and **Figure S9A, S9B**), but processing of proIL-1β to its active form in response to virus was completely abolished upon loss of caspase-1 or NLRP3 (**Figure 4C**); implicating that NLRP3 and caspase-1 mediate HCV-induced IL-1β maturation. Consistent with this observation, expression knockdown of ASC, the essential NLRP3 signaling-adaptor protein [35], similarly abrogated the processing of IL-1β (**Figure 4C** and **Figure S9C**). Thus, HCV induces the maturation of proIL-1β in macrophages through activation of the NLRP3 inflammasome.

HCV products have been shown to induce reactive oxygen species (ROS), while products of virus infection have been shown to drive sodium and potassium channel activities to mediate intracellular ion flux and pH change, each of which are implicated as cellular metabolic changes that induce the assembly of the NLRP3 inflammasome [36]–[39]. To define the virus-induced metabolic changes that might impart NLRP3 inflammasome activation upon cellular uptake of HCV, we pretreated cells with diphenyleneiodonium (DPI, ROS inhibitor) or glybenclamide (Glyben, inhibitor of potassium channels) and assessed processing of IL-1β. While ROS inhibition only affected IL-1β processing induced by HCV at high doses (**Figure S10**), IL-1β maturation was blocked in a dose-dependent manner by glyben treatment of cells (**Figure 4D**), implicating HCV-induced potassium efflux as a trigger of the NLRP3 inflammasome. Consistent with this notion, IL-1β maturation was blocked when cells were cultured in isotonic media containing 100 mM KCL to prevent intracellular potassium depletion while culturing in isotonic NaCl media did not alter HCV-induced IL-1β maturation (**Figure 4E**). These results reveal a conserved process of virus-induced inflammatory signaling in which HCV induction of potassium efflux leads to NLRP3 inflammasome activation. Taken together, our observations define the recognition of viral RNA by TLR7 and signaling via MyD88 to drive IL-1β mRNA expression with subsequent NLRP3 activation triggered by virus-induced potassium efflux as the mediators of HCV-induced IL-1β production in macrophages.

### Macrophage exposure to HCV induces inflammasome signaling of IL-1β and proinflammatory cytokine and chemokine expression

**Figure 5:**
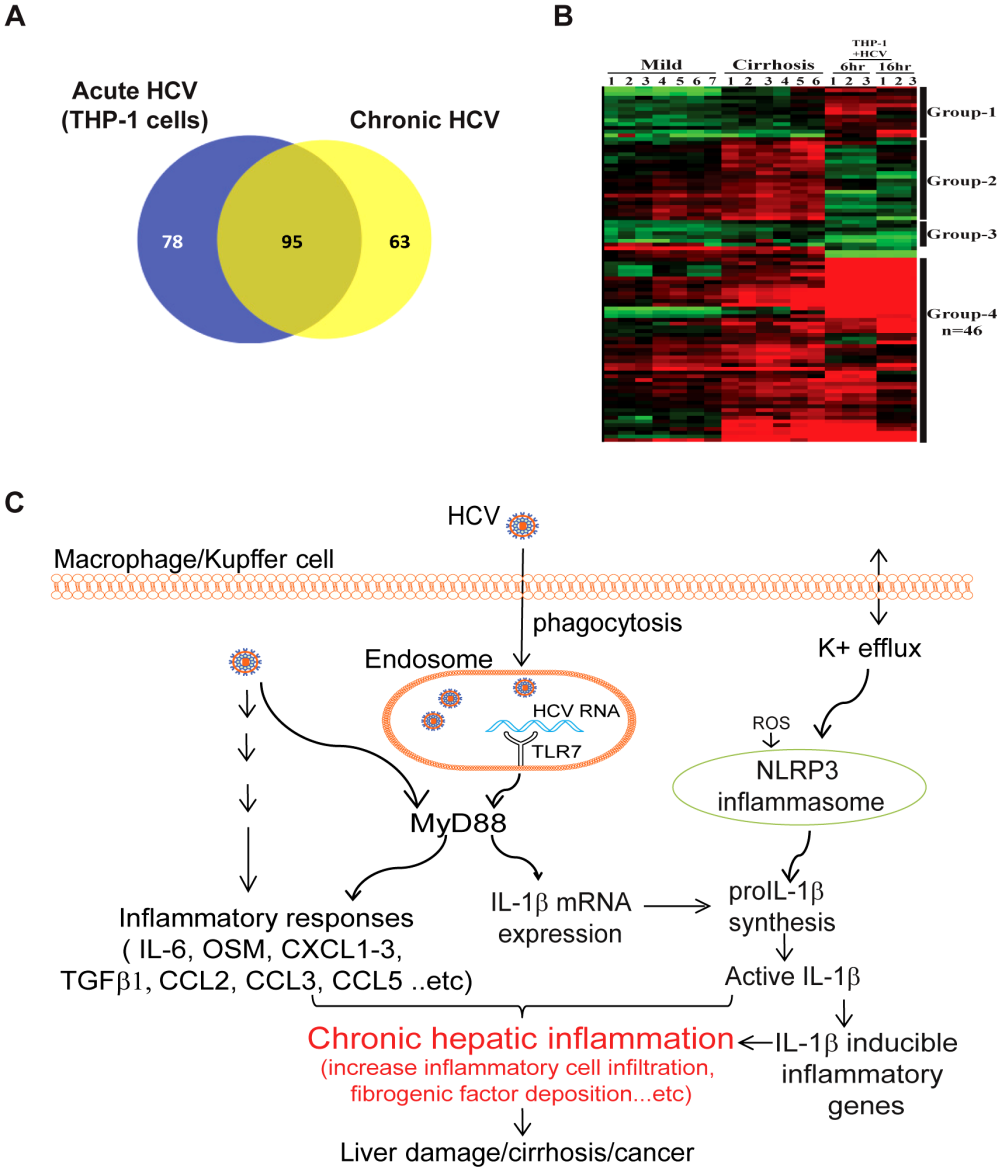
Macrophage exposure to HCV induces inflammasome signaling of IL-1β and proinflammatory cytokine and chemokine expression. (**A** and **B**) RNA-seq analysis to directly compare the transcription profile of THP1 cells after acute HCV (moi = 0.01) exposure for 6 and 16 hours and chronic hepatitis C patient liver staged by mild or severe disease. (**A**) The Venn diagram shows the number of differentially expressed genes (>1.5-fold change and FDR, 0.05) that were unique and common to THP-1 cells and chronic hepatitis C patient liver in the most highly represented KEGG pathways in both datasets, the cytokine-cytokine receptor signaling, and chemokine signaling pathways. (**B**) Hierarchical clustering analysis of differentially expressed genes common to both HCV-exposed THP-1 cells and chronic hepatitis C liver. Group-4 genes were expressed in both THP-1 cells and chronic hepatitis C liver (for full description, see Table S2). Group-4 genes and the expression key are shown at the right. See Methods for a description of bioinformatics analysis. (**C**) Model of hepatic inflammatory signaling during HCV infection. Sensing of HCV by hepatic macrophages triggers the induction of IL-1β and the inflammatory response. HCV-induced inflammasome activation is initiated by endosomal TLR7 engagement of viral RNA to drive the induction of IL-1β expression via MyD88. In addition, HCV triggers potassium efflux for NLRP3 activation to produce mature IL-1β. Secreted IL-1β induces the expression of a wide-range of proinflammatory mediators and stimulates an inflammatory response that confers liver disease during chronic infection.

Our study identifies macrophage-produced IL-1β induced by cell exposure to HCV as a central component initiating the inflammatory response linked with HCV pathogenesis, in which IL-1β produced by macrophages transmits an IL-1β-driven inflammatory response globally within liver tissue. To ascertain how initial inflammasome signaling in macrophages propagates into a hepatic inflammatory response, we conducted a direct comparative RNA-seq analysis of gene expression datasets from chronic hepatitis C patients to the RNA-seq transcriptome of THP1 cells during an acute time course after exposure to HCV. The comparison identified a bioset of commonly induced genes in the two most highly represented pathways, cytokine-cytokine receptor signaling, and chemokine signaling, of which a subset of genes associated with severe liver disease in HCV patients, including IL-1β and IL-1β-responsive proinflammatory products (**Figure 5A, 5B** and **Table S2**; see **Group 4**). These observations imply that in patients with chronic hepatitis C, Kupffer cells and/or infiltrating liver macrophages produce IL-1β, driving a hepatic response that includes the expression of a wide range of proinflammatory mediators of liver inflammation, fibrogenesis and disease.

## Discussion

Our findings reveal that IL-1β production and hepatic inflammation in HCV infection are linked and driven through virus-induced TLR7 and NLRP3 inflammasome signaling in liver macrophages. These observations support a model of hepatic inflammation induced by phagocytic uptake of HCV by resident macrophages/Kupffer cells to trigger IL-1β and drive expression of proinflammatory cytokines, with IL-1β expression associating with liver disease in patients with chronic hepatitis C infection (**Figure. 5C**). Importantly, macrophages are not a tropic cell for productive HCV infection. Our studies demonstrate that IL-1β is stimulated by HCV within hepatic macrophages in a manner that is independent of actual infection but mediated by phagocytic uptake of virus. As HCV circulates at high levels with monocytes in the bloodstream of patients with chronic hepatitis C, it is noteworthy that the phagocytic uptake of HCV was essential for induction of inflammasome activity in the macrophage model, and that undifferentiated monocytes do not phagocytose HCV. The requirement of phagocytic uptake of HCV for inflammasome stimulation provides an important checkpoint against systemic IL-1β production by blood monocytes under conditions of HCV viremia, thus limiting inflammatory signaling to differentiated tissue macrophages within the local hepatic environment. Indeed, phagocytic uptake of HCV and non-self-recognition of viral RNA is sufficient to trigger IL-1β production from hepatic macrophages. Although RIG-I-deficient hepatoma cells have been shown to produce low levels of IL-1β during HCV infection [40], we found that HCV infection of immune-competent hepatoma cells and primary hepatocytes does not trigger appreciable production of IL-1β. These observations reveal an important regulatory feature of IL-1β production from different cell sources, as hepatocytes comprise the liver parenchyma and are constantly exposed to blood-borne microbes like HCV where a wide release of IL-1β could induce tissue toxicity. Rather, macrophages, including Kupffer cells, serve a specialized role to sample the local environment via phagocytosis and selectively render inflammatory signaling upon pathogen identification, such as TLR7/MyD88-dependent signaling upon recognition of internalized HCV RNA.

HCV products induce intracellular reactive oxygen species and ion flux, both of which trigger the NLRP3 inflammasome during virus infection, whereas potassium efflux is essential for inflammasome signaling by HCV. Our study also reveals that in macrophages, HCV RNA is engaged by both RIG-I/MAVS-dependent and TLR7/MyD88-dependent PRR pathways to induce innate immune (IFN-β) and inflammatory (IL-1β) signaling, respectively. In this respect, the intracellular compartmentalization of HCV upon macrophage uptake could expose virion components such as HCV RNA to endosomal TLR7 for PRR engagement [41]. These observations also reflect a key difference from hepatocytes in which HCV RNA replication products of infection accumulate at endoplasmic reticulum membrane-associated cytosolic replication sites independent of endosomal TLRs but accessible by RIG-I [42], thus amenable to triggering IFN-β defenses but not IL-1β production. Our RNA transfection experiments of THP-1 cells deliver HCV RNA into both endosomal and cytoplasmic compartments for signaling and reveal the dual potential of the viral RNA to be sensed by both RIG-I and TLR7 within a compartment-dependent context within macrophages. Thus, HCV products impart intracellular signaling and metabolic changes that drive IL-1β production from macrophages. In the liver, this process is likely mediated by Kupffer cells, though it may also occur in lymphoid tissue and other sites exposed to HCV that are populated with macrophages or other phagocytic cells.

A variety of viruses have been shown to activate the NLRP3 inflammasome, including influenza virus and *flaviviruses* (West Nile virus and Japanese encephalitis virus). Like other *flaviviruses*, HCV has a positive stranded RNA genome. Whereas NLRP3 inflammasome activation and production of IL-1β during infection by West Nile virus or Japanese encephalitis virus is essential for proper immune induction and virus control [11], [12], NLRP3 activation of IL-1β production by HCV associates markedly with immunopathogenesis from hepatic inflammation. This relationship is further evident by our RNA-seq analysis, which revealed that IL-1β and IL-1β-driven genes are associated with severe liver disease. In infected hepatocytes, HCV subverts innate immune signaling by targeting and suppressing the RIG-I pathway [21]. Innate immune evasion supports chronic HCV infection and viremia that lends to macrophage uptake of HCV and signaling by TLR7 and the NLRP3 inflammasome. The linkage of these processes of innate immune evasion/persistent viral replication and viremia/IL-1β production and response thus mediates a cycle of chronic inflammatory stimulation that underlies liver disease in HCV infection.

We found that, following virus uptake in macrophages, HCV proteins are transiently produced but then decay. HCV proteins have been shown to stimulate ROS accumulation and regulate ion efflux. Moreover, the HCV p7 protein is a transmembrane cation channel and a member of the viroporin family whose actions can drive ion flux that could impart NLRP3 inflammasome activation during HCV infection [43], [44]. Thus, while HCV RNA triggers inflammasome “signal one” via TLR7, the transient production of p7 and other HCV proteins may provide stimulus for “signal two”, including potassium flux that induces NLRP3 activation for IL-1β maturation and secretion. The constant induction of IL-1β and proinflammatory genes by hepatic macrophages in chronic hepatitis C patients would then serve to recruit immune cells to the liver and augment the inflammatory state resulting in liver fibrosis and cirrhosis (see **Figure 5C**). Moreover, recent studies show that HCV uptake by hepatic plasmacytoid dendritic cells and by Kupffer cells can induce their production and secretion of IFN-β, thus driving ISG expression and an inflamed state in the local surrounding hepatocytes [23], [26], [45]. Whereas HCV infection is treated with IFN-based therapy [46] and direct-acting antiviral drugs [47], the local ISG expression from these hepatic myeloid cells can render a state of innate immune tolerance to attenuate the antiviral actions of IFN. As a result, IFN treatment is only partially effective in suppressing HCV [26]. Moreover, it has been revealed that the IFN response can antagonize inflammasome signaling [48] while IL-1β can enhance the expression of specific ISGs to impart increased effectiveness of IFN actions [11]. Thus, whereas effective antiviral therapy for HCV may actually reduce liver inflammation [49], this process could involve signaling crosstalk between IFN and IL-1β that overall enhances IFN actions [11]. Understanding how such crosstalk imparts restriction of HCV infection and effective antiviral immune responses should reveal novel IFN/IL-1β interactions that balance liver inflammation and immune actions for the control of liver disease. Interventions that target IL-1β or inflammasome components [50]–[52] could thus serve as therapeutic applications to mitigate HCV-induced hepatic inflammation and disease, particularly where antiviral agents have failed.

## Materials and Methods

### Ethics statement

Sera from normal donor or patients with chronic hepatitis C and liver biopsy tissue from patients with chronic hepatitis C or controls were obtained with written informed consent and approval from the human subjects institutional review board (IRB) of University of Washington, University of Utah, Harvard Medical School and Singapore institute for clinical sciences.

### Patient samples

Chronic hepatitis C patient sera were obtained from patients infected with HCV genotype-1b who were treatment naïve and exhibited liver inflammation with various stages of fibrosis. Liver biopsies for immune-staining analysis studies were from patients with chronic HCV-1b infection who had previously undergone standard of care IFN/ribavirin therapy and were classified as non-responders with mild liver disease. For RNA-seq studies, patient liver specimens were analyzed by RNA sequencing and included normal (unused donor liver; n = 6), mild disease chronic hepatitis C (percutaneous liver biopsies with mild inflammation [Metavir grade 1–2] and no fibrosis; n = 7) and severe disease chronic hepatitis C (liver explants from patients with chronic hepatitis C and cirrhosis undergoing liver transplantation; n = 6). Biopsy specimens were from both men and women and were obtained under IRB approval. All samples were frozen in liquid nitrogen in RNAase free tubes immediately after collection and stored at −80°C until they were processed for RNA extraction and analysis. For cytometric analysis, healthy human liver-associated mononuclear cells were collected from livers of living donors (n = 2) after portal flush using cold (4°C) preservation solution following removal of right liver lobes. Collection was performed according to the standard protocol preceding liver transplantation [53]. Subsequently, liver resident/intrasinusoidal mononuclear cells were isolated by density gradient centrifugation on Ficoll-Hypaque. The CD14+ cells (negative for CD3, CD7, CD56, CD19 and CD20) present in this cell population expressed CD68 and Cd11b to levels similarly to the CD14+ cells purified from homogenized livers and therefore are defined as “Kupffer cells” [54]. Total purified cells from the liver wash-out were plated in a 96-well round bottom plate, treated with Brefeldin A (10 µg/ml) and stimulated either with LPS (1 µg/mL), HCV-containing (MOI 0.1) or mock supernatant for 6 hours. Cells were then surface stained for CD14 (clone M5E2, BD) and CD16 (clone 3G8, Biolegend) for monocytes, as well as for CD3 (clone HIT3a, Biolegend), CD7 (clone 4H9, eBioscience), CD56 (clone HCD56, Biolegend), CD19 (clone HIB19, Biolegend) and CD20 (clone 2H7, Biolegend) for the lineage positive cells, i.e., T-NK-B cells. Fluorescence and size data from stained cells were acquired using a Becton Dickenson LSR II flow cytometer and analyzed using FACS Diva (BD) or FlowJo (Tree star) software.

### Reagents and antibodies

Anti-IL-1β was purchased from R&D systems and from Cell Signaling. Anti-caspase-1 and anti-CD68 antibodies were purchased from Santa Cruz biotechnology. Anti-MyD88 was purchased from Enzo Life Science. Anti-human CD81 (JS-81) was purchased from BD pharmingen. Anti-MAVS antibody was from Abcam, and anti-ASC was from MBL technologies. Anti-human ISG56 rabbit polyclonal antibody is a gift from Dr. G. Sen. Human IL-1β, IFNβ, NLRP3, TLR7 and GAPDH oligonucleotide PCR primers were purchased from Qiagen (Superarray bioscience). For HCV protein detection, serum A kindly provided by Dr. William Lee was used as an immunoblot probe for viral proteins. Mouse IgG1 (MOPC21), mission lentiviral transduction particles (Non-targeting, caspase-1, NLRP3, ASC, MAVS, TLR7 or MyD88-specific), adenosine 5′-triphsophage (ATP) disodium salt solution, Phorbol 12-Myristate 13-acetate, LPS, cytochalasin D, bafilomycin A, diphenyleneiodonium chloride and glybenclamide were each purchased from Sigma. Synthetic dsRNA Poly (I): Poly(C) was from GE Healthcare Life. TransIT-mRNA transfection kit was from Mirus and IL-1β ELISA kit was from Biolegend.

### Cell culture

THP-1 cells were purchased from ATCC and grown in complete RPMI-1640 medium containing 10% fetal bovine serum, antibiotics, L-glutamine, pyruvate, and non-essential amino acid. THP-1 cells were differentiated by treatment with 20–40 nM of PMA overnight at 37C. Huh7 [55], Huh7.5, and immortalized human hepatocytes (PH5CH8 [56] and IHH- a gift from Dr. R. Ray, St Louis University, St. Louis, MO.) were grown in Dulbecco's Modified Eagle's Medium *(DMEM)* supplemented with 10% fetal bovine serum, antibiotics, L-glutamine, pyruvate, non-essential amino acid and Hepes. Monocyte-derived macrophages were generated from peripheral blood mononuclear cells (PBMCs) obtained from healthy individuals under IRB approval. PBMCs were isolated as described previously [57] and macrophages were cultured using plastic adherence as described elsewhere [58] and cultured in complete RPMI. All cells were incubated at 37C with 5% CO_2_.

### HCV propagation

An HCV JFH-1 genotype 2a infectious clone was produced from synthetic cDNA constructed from the published sequence of original JFH-1 clone [59]. This virus, termed HCV-SJ (**S**ynthetic **J**FH1) was then produced directly from in vitro-transcribed RNA transfected into Huh7.5 cells, and resulting virus was passaged for culture adaptation. The culture-adapted HCV-SJ will be described in a subsequent report. For infection studies, HCV-SJ was grown in Huh7.5 cells, concentrated 100× using Centricon 100,000 MW cut-off filters (Millipore, Billerica, MA) and titered to determine focus forming units (ffu)/ml using a Huh7 cell-based FFU assay. Differentiated THP-1 cells were treated with HCV multiplicity of infection [moi = 0.01–1 based on Huh7 focus forming units (ffu)]. We note that preparations of HCV quantified by the FFU method contain both infectious and noninfectious particles with the latter typically represented in 100 to 1000-fold excess over the former and thus likely contribute to the THP1 cell response to HCV [21], [60], [61]. HCV virions were purified under sucrose cushion by ultracentrifugation. Immortalized primary hepatocytes and hepatoma cells were infected with HCV at moi = 0.1. Ultraviolet light inactivation was achieved using the spectrolinker UV crosslinker to irradiate HCV-containing cell culture media. The media containing UV-inactivated virus was then used to infect hepatocytes or to stimulate THP-1 cells.

### Quantitative Real Time PCR (qRT-PCR)

Total RNA was extracted from cultured cells using the Qiagen RNeasy mini kit. qRT-PCR was performed as previously described [62]. Specific qRT-PCR primers for HCV were: probe 238–267: 5′ CCCGCAAGACTGCTAGCCGAGAGTGTTGG 3′, forward primer 98–116:5′ GAGTGTCGTGCAGCCTCCA3′, reverse primer 313–294: 5′CACTCGCAAGCACCCTATCA3′, the probe contains 5′ 6-FAM and 3′ TAMRA-sp modification. RNA amplification was conducted from 50 ng cDNA conducted in 25 µl reaction mixture in optical 96-well plates. The reactions were performed under the following conditions: 30 min at 48C, 1 cycle at 95C for 10 min, 40 cycles at 95C for 15 seconds and 1 min at 60C and dissociation curve (95C 1min, 65C 2 min and 65C–95C at 2/sec).

### Enzyme-linked Immunosorbent Assay

IL-1β ELISA was run according to the manufacture's protocol (Biolegend).

### Immunoblot analysis

Cell lysates were recovered from control or HCV-stimulated samples via cell lysis in RIPA buffer (50 mM Tris-HCl pH 7.4, 1% Triton-x, 0.25% Na-deoxycholate, 150 mM NaCl, and 1 mM EDTA). 20–30 µg of protein was subjected to 15% SDS-PAGE followed by immunoblot assay using the indicated antibodies as described [30].

### Immunohistochemical staining and confocal microscopy

Slide-mounted paraffin-embedded biopsy tissue was deparaffinized, subjected to antigen retrieval, and immunostained with the indicated antibodies exactly as described previously [63].After staining, cover slips were mounted on slides using Prolong Gold mounting medium (Invitrogen). Immunostained tissues were visualized using a Nikon Eclipse TE2000 inverted microscope with the Nikon C1 laser scanning confocal module attached to a 10 mW Argon laser, 1 mWHeNe laser, and a 5 mWHeNe laser emitting light at 477 nM, 543 nM, and 633 nm wavelength, respectively. Confocal digital images were collected as 0.2 µM optical sections and were processed using Nikon EZ-C1 Software v.3.40. Multiple images were collected for each sample analyzed. The primary antibodies used were anti- IL-1β and anti-CD68.

### *In vitro* transcription of HCV RNA

In vitro transcribed full length HCV RNA (JFH-1), polyU/UC RNA, and X region RNA was prepared from linearized plasmid DNA encoding the synthetic JFH1 HCV 2a genome or specific subgenomic regions using Ambion Mega Script and purified free of DNA exactly as described [30].

### RNA sequencing and transcriptomics analysis

For RNA sequencing (RNA-seq) and associated bioinformatics, total RNA was purified from cells or clinical biospecimens using Trizol (Invitrogen, USA) as described previously; and only RNA specimens yielding a Bioanalyzer (Agilent, USA) RNA integrity number (RIN) of ≥8.0 were used [64]. cDNA libraries were prepared from poly(A) selected mRNA following Illumina RNA-seq protocols [65]. Single 50 bp RNA-Seq reads were obtained using Illumina HiSeq 2000 protocols and analyzed following previous studies [66]. A total of 20–29 million 50 bp reads were obtained for all chronic hepatitis C and control biospecimens and 16–26 million 50 bp reads for all THP-1 cell samples. Bioinformatics analysis of the RNA-seq data included adjustments for the depth of sequencing [66]. Sequencing reads were aligned to the February 2009 human reference genome (GRCh37/hg19), with July 2011 updates, using Novoalign software. The USeqOverdispersedRegionScanSeqs (ORSS) application was used to count reads intersecting exons of each annotated gene and score them for differential expression in each sample. Scores were controlled for multiple testing and ranked by false discovery rate (FDR) and normalized ratio. Genes designated as significantly differentially expressed had an untransformed FDR of <0.05 (<5 false positives per 100 observations) and normalized change of ≥1.5 fold relative to controls. Hierarchical cluster analysis to produce heat maps of differentially expressed genes was done with Cluster 3.0 software and visualized using Java TreeView [67]. Red = increased expression and green = reduced expression.

### Endotoxin testing

LPS contamination of cell extracts was tested by gel clot assay using *Limulus* Amoebocyte Lysate (LAL) Pyrotell from CAPE COD, Inc. according to manufacturer's recommendations.

### RNA transfection

Differentiated THP-1 cells were transfected using the Mirus reagent following the company's protocol (Mirus).

### Lentiviral shRNA transduction

Undifferentiated THP-1 cells were transduced with 1–3×10∧6 lentiviral particles encoding non-targeting control or gene-specific shRNA in 48 well plates. 24 hrs post transduction, selection media was added (RPMI containing puromycin at 1 µg/ml). The cells were then maintained in selection media. For assessment of mRNA knockdown, THP1 cells were differentiated with PMA prior to use in experiments.

#### Statistical analysis

Statistical analyses were conducted using unpaired student *t-test* and GraphPad prism software.

## Supporting Information

Figure S1 Validation of chemokine and cytokine gene expression induced by IL-1β treatment of THP-1 cells. Differentiated THP-1 cells were treated with 100 ng/ml of recombinant IL-1β for 6 or 24 hr. RNA was extracted and subjected to qRT-PCR analysis of gene expression.
(TIF)

Figure S2 (**A**) Immortalized hepatocytes (IHH and PH5CH8) and hepatoma Huh7 cells were infected with HCV at moi of 0.1 then IL-1β mRNA expression (upper panel) and protein secretion (lower panel). (**B**) Immunoblot examining the expression of inflammasome components in infected hepatocytes and hepatoma cells.
(TIF)

Figure S3 Quantification of CD68+ hepatic macrophages/Kupffer cells determined from healthy donor and chronic hepatitis C patient liver sections. Liver sections were immuno-stained with anti-CD68 antibody and analyzed by confocal microscopy. Bars show average cell number and standard error from manual counting of at least three independent fields from tissues of three patients each.
(TIF)

Figure S4 Huh7.5 cells were infected with either viable or UV-inactivated HCV at moi = 0.1. 48 hr later, the cells were harvested, and RNA and protein were extracted for qRT-PCR analysis (**A**) and viral protein abundance by immunoblot assay (**B**) antiserum from an HCV patient. Positions of viral proteins are indicated.
(TIF)

Figure S5 Immunoblot showing viral proteins in HCV-infected Huh7.5 cells. Cells were infected with HCV-containing supernatant (HCV sup) or purified HCV virion from sucrose gradient ultracentrifugation of infectious supernatant. Cells were infected with equivalent 0.1 focus forming units (ffu) of either infectious HCV sup or purified HCV virions for 1 hr. 48 hr later, the cells were harvested and extracts were subjected to immunoblot analysis using HCV patient antiserum. Positions of viral proteins are indicated.
(TIF)

Figure S6 (**A**) HCV RNA in THP-1 cells. Differentiated THP-1 cells were pre-treated either with anti-CD81 or isotype control for 1 hour at the indicated concentrations. Cells were then washed and incubated with HCV (moi = 0.01 based on Huh7 ffu) in the presence or absence of anti-CD81 or isotype control antibody at the same concentration. After 3 hours, cell lysates were harvested, and RNA was extracted and subjected to qRT-PCR analysis for determination of HCV RNA copy number as defined by qRT-PCR of standard HCV RNA template control. (**B**) Differentiated THP-1 cells were pretreated with bafilomycin (2.5 µM) or cytochalasin D (10 µM) for 1 hr and then exposed to HCV in the presence or absence of continued drug treatment. Cell lysates were harvested 3 hrs later, and RNA was extracted and subjected to qRT-PCR analysis for determination of HCV RNA copy number as defined by qRT-PCR of standard HCV RNA template control.
(TIF)

Figure S7 Immunoblot assay of MyD88 (**A**) or MAVS (**B**) abundance in THP1 cells expressing non-targeting control shRNA or shRNA specific to MyD88 (**A**) or MAVS (**B**). Cells were infected with Sendai virus (SenV) for 24 hr prior to harvest. ISG56 and tubulin expression were respectively monitored as innate immune response gene and protein loading controls. This immunoblot confirms the functional knockdown of MAVS as ISG56 is a MAVS-dependent gene in this context and its production was completely abolished in shMAVS-expressing cells.
(TIF)

Figure S8 IL-1β and TLR7 expression in THP1 cells expressing non-targeting control shRNA or shRNA targeting TLR7. (**A**) Cells were mock-treated or treated with HCV (moi = 0.01 Huh7 ffu) for 6 hr and RNA extracted for qRT-PCR analysis of IL-1β mRNA expression. (**B**) TLR7 mRNA levels were assessed 6 hr after transfection reagent alone or HCV polyU/UC RNA transfection.
(TIF)

Figure S9 Immunoblot showing the levels of caspase-1 (**A**), NLRP3 (**B**) and ASC (**C**) in cells expressing specific knockdown shRNA in THP-1 cells transduced with lentiviral particles as compared to non-targeting control.
(TIF)

Figure S10 Differentiated THP-1 cells were, from left to right, mock treated, treated with HCV (moi = 0.01 based on Huh7 ffu) or pretreated with 0.3,0.6, 1.25, 2.5, 5, 10, 20, 40, 80 µM Diphenyleneiodonium chloride (DPI) for 1 hr followed by HCV treatment. 3 hr later cells were harvested and extracts subjected to immunoblot analysis for mature IL-1β and tubulin.
(TIF)

Table S1 Differentially expressed genes in liver biopsy specimens from chronic hepatitis C patients with mild (no fibrosis) and severe (cirrhosis) liver disease represented in (**Figure 1A**) Group-1 shows genes reduced in expression or “down-regulated” in association with HCV infection compared to control liver; group-2 shows genes with increased expression or “up-regulated” in HCV infection (mild and severe disease) compared to control liver; group-3 shows genes highly up-regulated in association with severe disease only compared to control liver; group-4 shows genes highly down-regulated in mild but not in severe disease patient livers.
(TIF)

Table S2 Differentially expressed genes in chronic hepatitis C liver specimens with mild (no fibrosis) and severe (cirrhosis) disease or in THP-1 cells exposed to HCV represented in (**Figure 5B**). Group-1 shows genes up-regulated in HCV-exposed THP-1 cells only, as compared to mock-treated THP1 control cells; group-2 shows genes expressed in association with severe liver disease only but not in THP-1 cells; group-3 shows genes down-regulated in both hepatitis C liver and HCV-treated THP-1 cells; group-4 shows genes commonly expressed in both hepatitis C liver and HCV-treated THP-1 cells. Gene expression, as measured by RNA-seq analysis, in hepatitis C liver specimens was compared with control liver specimens. Gene expression in HCV-treated THP1 cells was compared with mock-treated THP1 cells.
(TIF)
