# Supplementary figures and images for: IL-1β Production through the NLRP3 Inflammasome by Hepatic Macrophages Links Hepatitis C Virus Infection with Liver Inflammation and Disease
Source: PLoS Pathog. 2013 Apr 25;9(4):e1003330. doi: 10.1371/journal.ppat.1003330 (PMC3635973; doi:10.1371/journal.ppat.1003330)

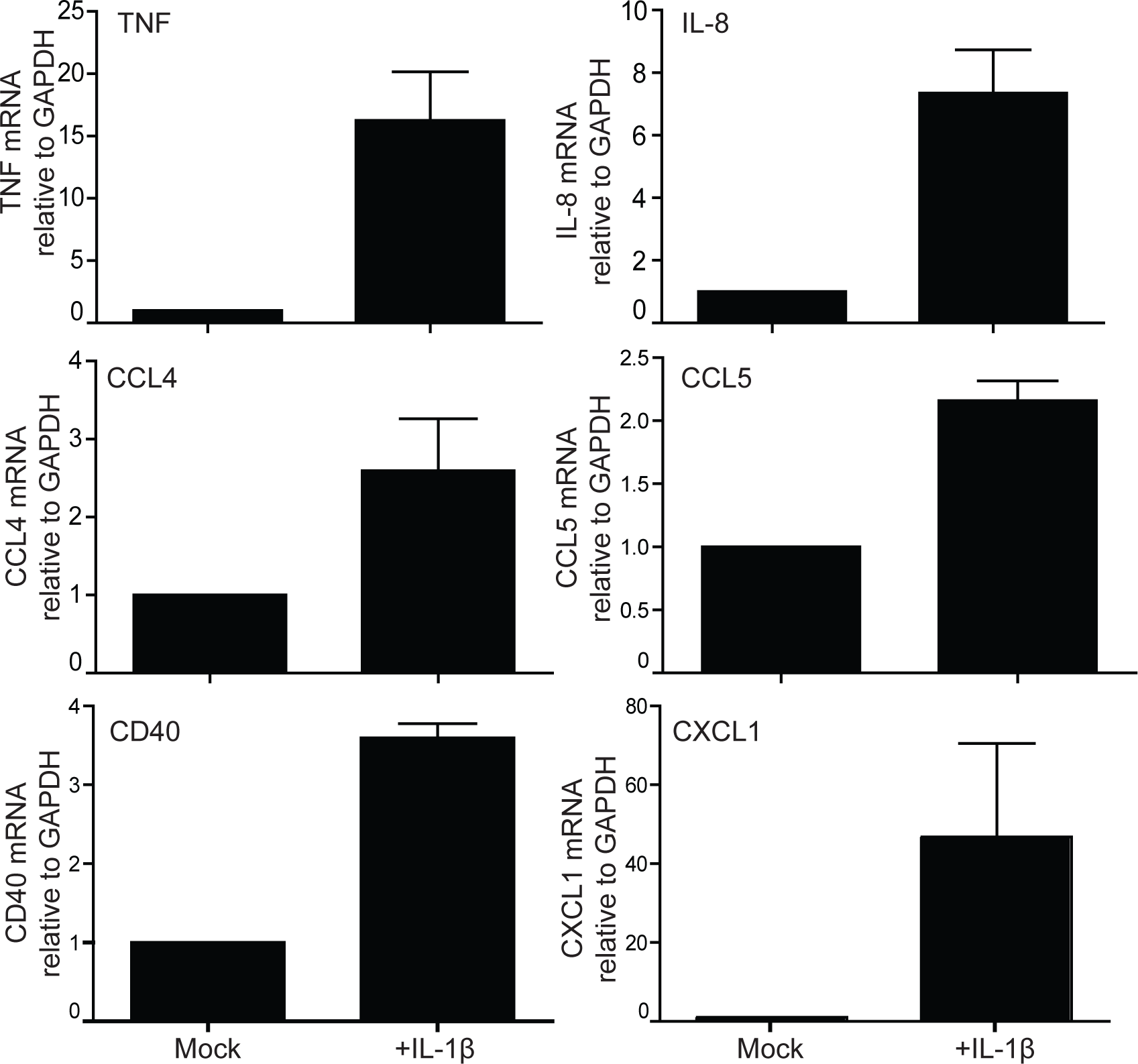

Supplement: Figure S1 — Validation of chemokine and cytokine gene expression induced by IL-1β treatment of THP-1 cells. Differentiated THP-1 cells were treated with 100 ng/ml of recombinant IL-1β for 6 or 24 hr. RNA was extracted and subjected to qRT-PCR analysis of gene expression. (TIF) [file ppat.1003330.s001.tif]

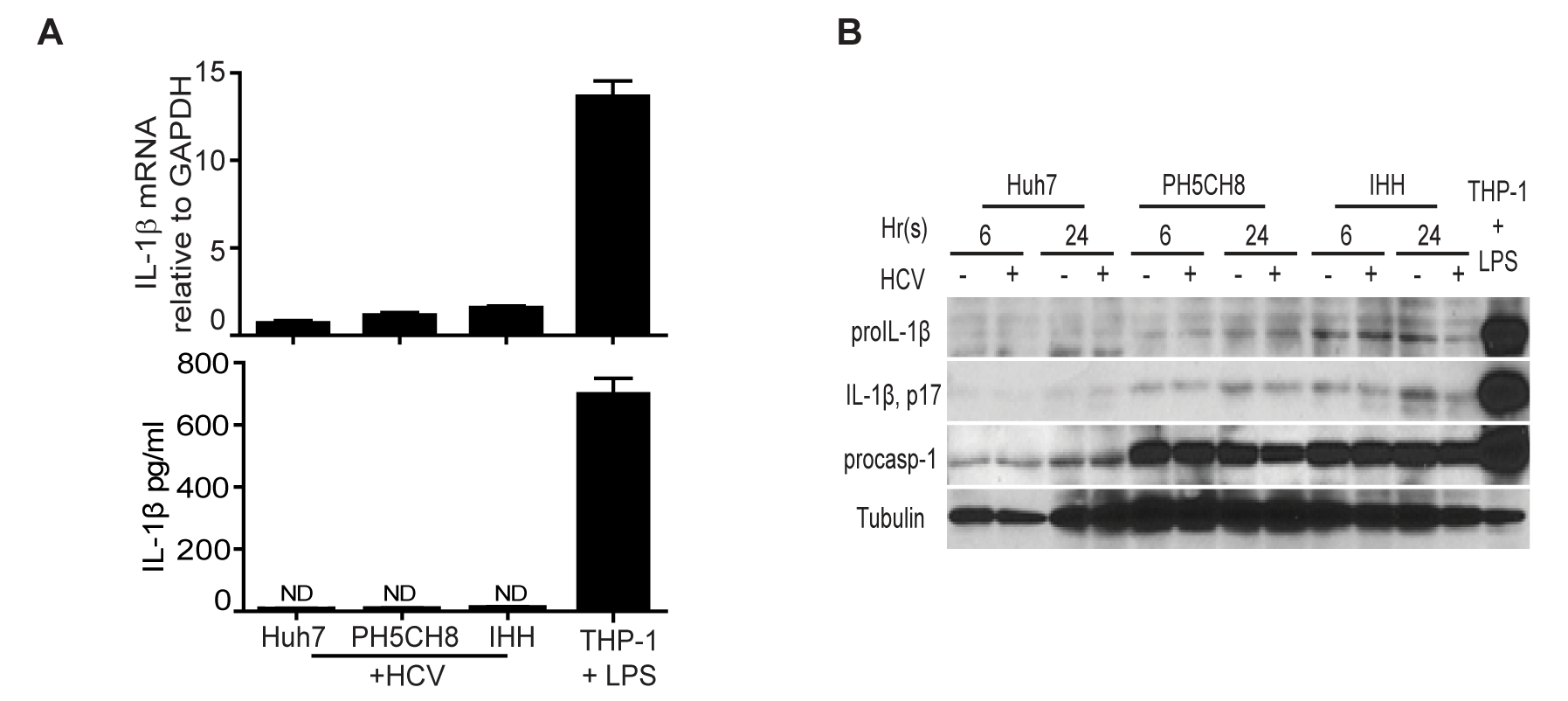

Supplement: Figure S2 — (A) Immortalized hepatocytes (IHH and PH5CH8) and hepatoma Huh7 cells were infected with HCV at moi of 0.1 then IL-1β mRNA expression (upper panel) and protein secretion (lower panel). (B) Immunoblot examining the expression of inflammasome components in infected hepatocytes and hepatoma cells. (TIF) [file ppat.1003330.s002.tif]

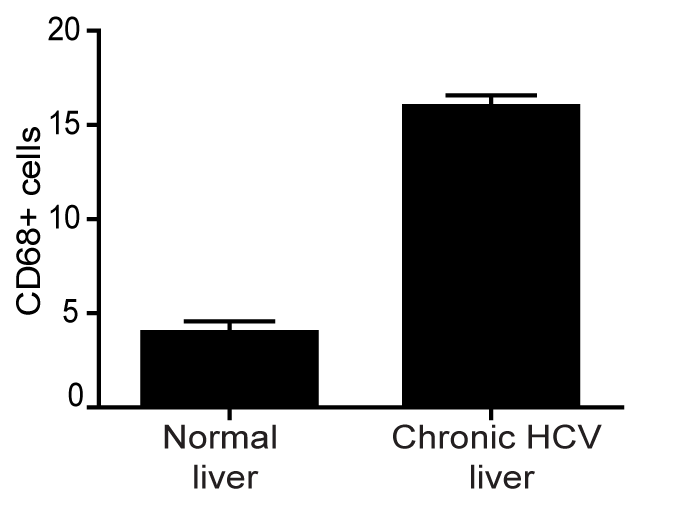

Supplement: Figure S3 — Quantification of CD68+ hepatic macrophages/Kupffer cells determined from healthy donor and chronic hepatitis C patient liver sections. Liver sections were immuno-stained with anti-CD68 antibody and analyzed by confocal microscopy. Bars show average cell number and standard error from manual counting of at least three independent fields from tissues of three patients each. (TIF) [file ppat.1003330.s003.tif]

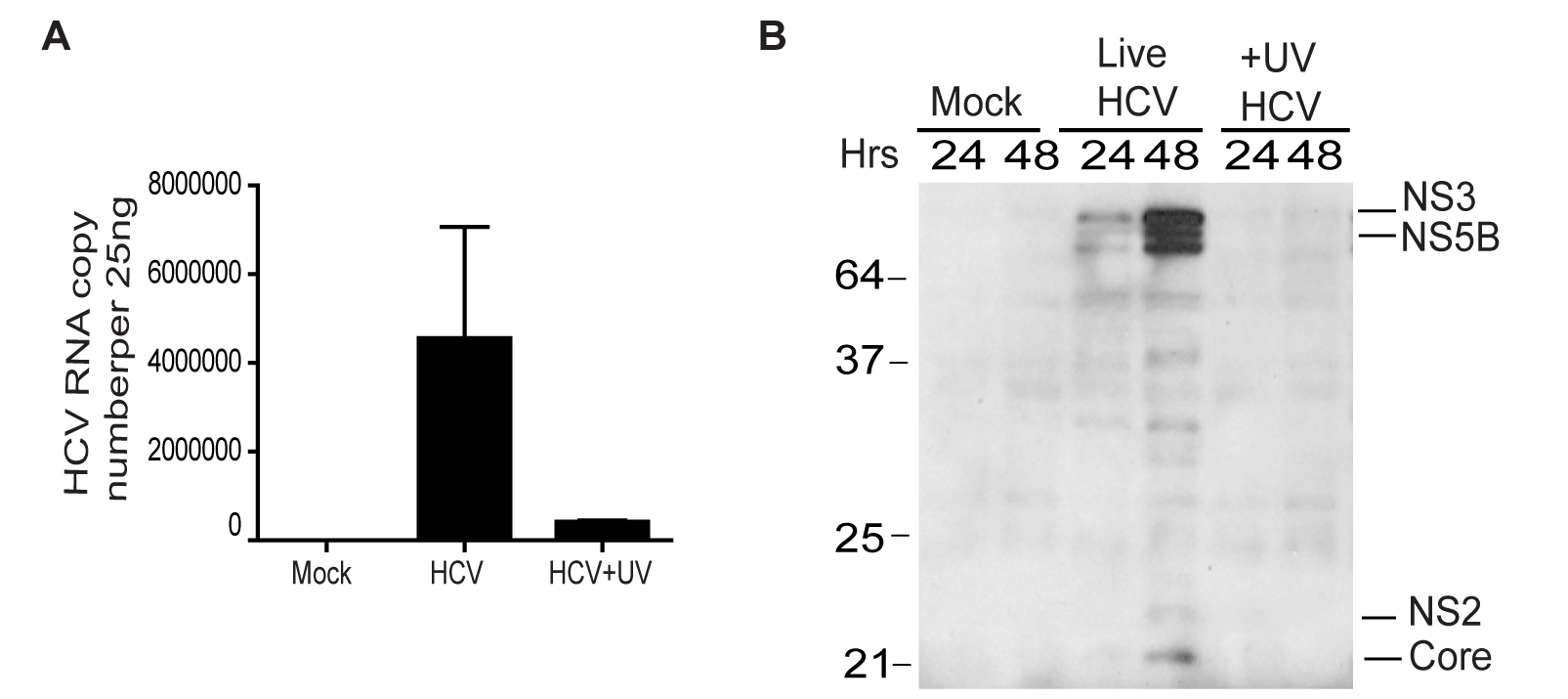

Supplement: Figure S4 — Huh7.5 cells were infected with either viable or UV-inactivated HCV at moi = 0.1. 48 hr later, the cells were harvested, and RNA and protein were extracted for qRT-PCR analysis (A) and viral protein abundance by immunoblot assay (B) antiserum from an HCV patient. Positions of viral proteins are indicated. (TIF) [file ppat.1003330.s004.tif]

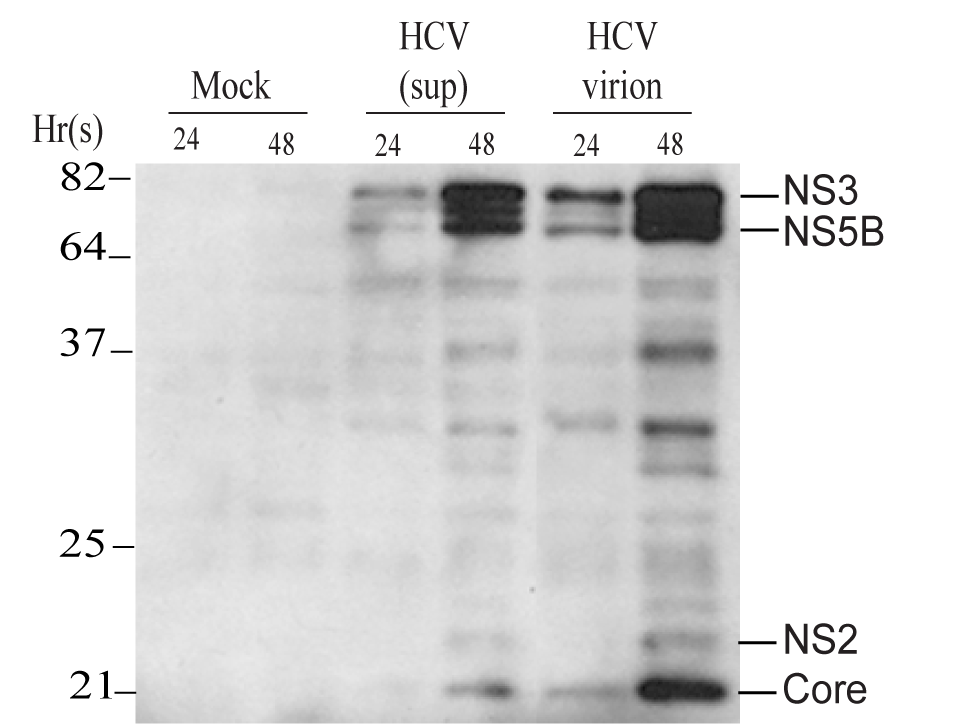

Supplement: Figure S5 — Immunoblot showing viral proteins in HCV-infected Huh7.5 cells. Cells were infected with HCV-containing supernatant (HCV sup) or purified HCV virion from sucrose gradient ultracentrifugation of infectious supernatant. Cells were infected with equivalent 0.1 focus forming units (ffu) of either infectious HCV sup or purified HCV virions for 1 hr. 48 hr later, the cells were harvested and extracts were subjected to immunoblot analysis using HCV patient antiserum. Positions of viral proteins are indicated. (TIF) [file ppat.1003330.s005.tif]

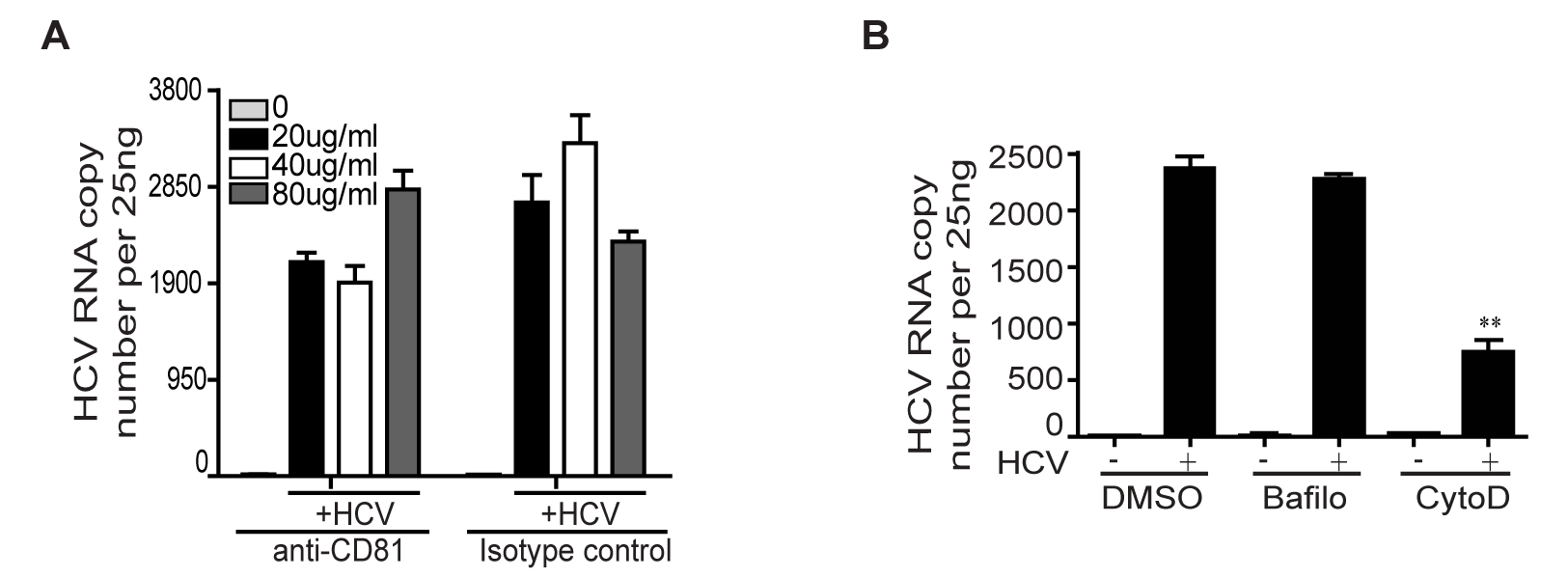

Supplement: Figure S6 — (A) HCV RNA in THP-1 cells. Differentiated THP-1 cells were pre-treated either with anti-CD81 or isotype control for 1 hour at the indicated concentrations. Cells were then washed and incubated with HCV (moi = 0.01 based on Huh7 ffu) in the presence or absence of anti-CD81 or isotype control antibody at the same concentration. After 3 hours, cell lysates were harvested, and RNA was extracted and subjected to qRT-PCR analysis for determination of HCV RNA copy number as defined by qRT-PCR of standard HCV RNA template control. (B) Differentiated THP-1 cells were pretreated with bafilomycin (2.5 µM) or cytochalasin D (10 µM) for 1 hr and then exposed to HCV in the presence or absence of continued drug treatment. Cell lysates were harvested 3 hrs later, and RNA was extracted and subjected to qRT-PCR analysis for determination of HCV RNA copy number as defined by qRT-PCR of standard HCV RNA template control. (TIF) [file ppat.1003330.s006.tif]

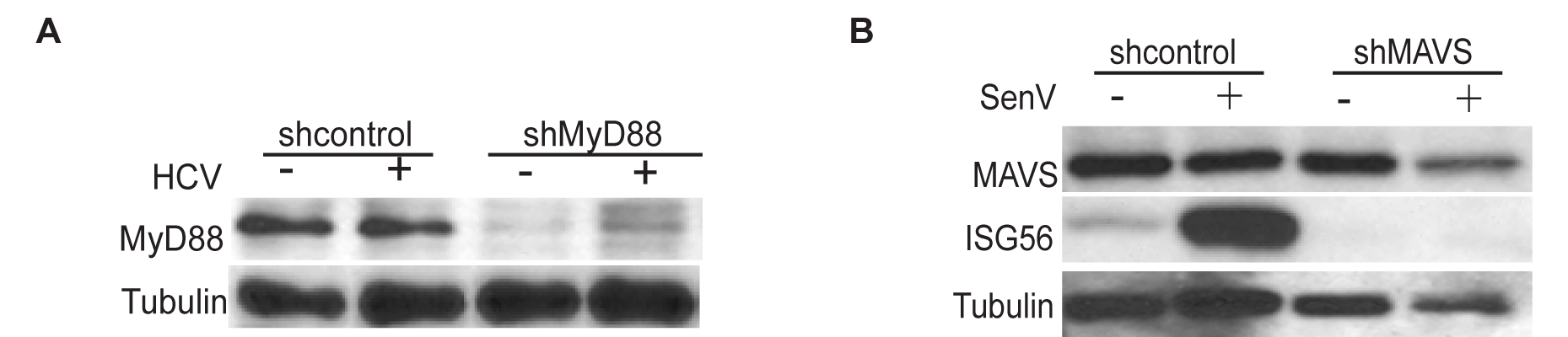

Supplement: Figure S7 — Immunoblot assay of MyD88 (A) or MAVS (B) abundance in THP1 cells expressing non-targeting control shRNA or shRNA specific to MyD88 (A) or MAVS (B). Cells were infected with Sendai virus (SenV) for 24 hr prior to harvest. ISG56 and tubulin expression were respectively monitored as innate immune response gene and protein loading controls. This immunoblot confirms the functional knockdown of MAVS as ISG56 is a MAVS-dependent gene in this context and its production was completely abolished in shMAVS-expressing cells. (TIF) [file ppat.1003330.s007.tif]

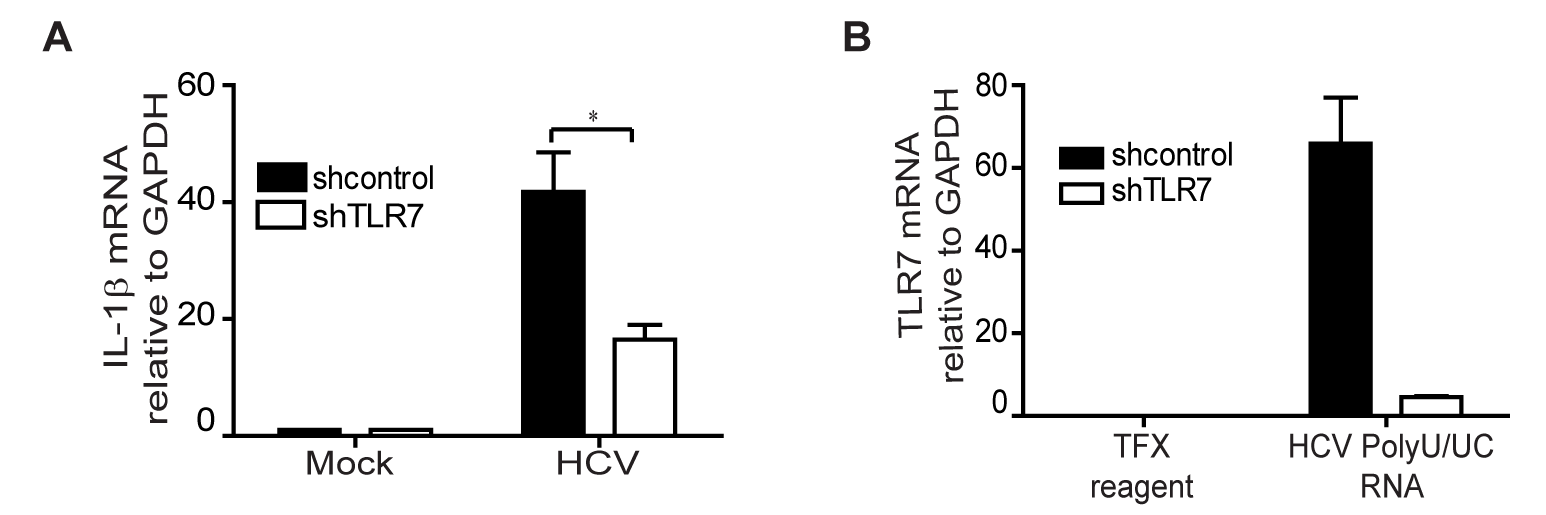

Supplement: Figure S8 — IL-1β and TLR7 expression in THP1 cells expressing non-targeting control shRNA or shRNA targeting TLR7. (A) Cells were mock-treated or treated with HCV (moi = 0.01 Huh7 ffu) for 6 hr and RNA extracted for qRT-PCR analysis of IL-1β mRNA expression. (B) TLR7 mRNA levels were assessed 6 hr after transfection reagent alone or HCV polyU/UC RNA transfection. (TIF) [file ppat.1003330.s008.tif]

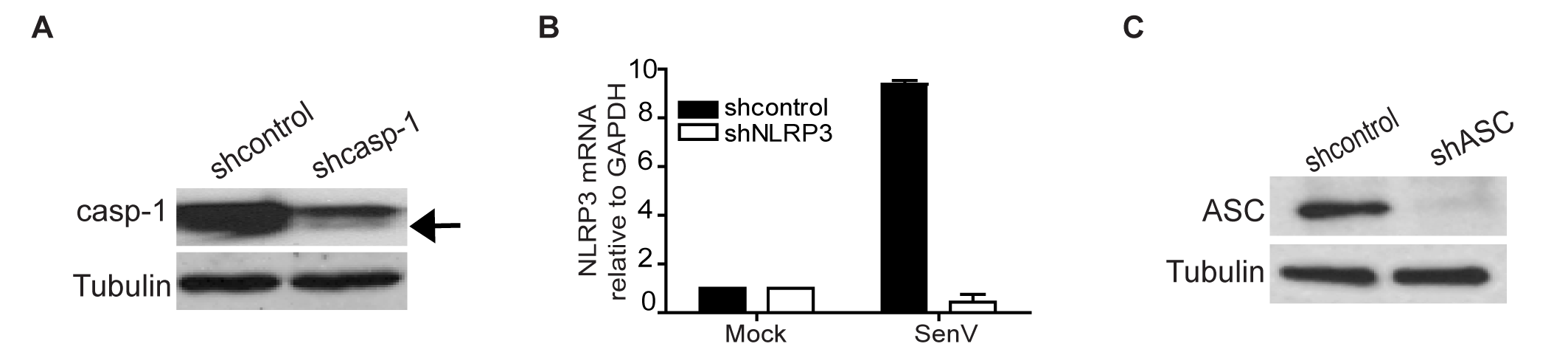

Supplement: Figure S9 — Immunoblot showing the levels of caspase-1 (A), NLRP3 (B) and ASC (C) in cells expressing specific knockdown shRNA in THP-1 cells transduced with lentiviral particles as compared to non-targeting control. (TIF) [file ppat.1003330.s009.tif]

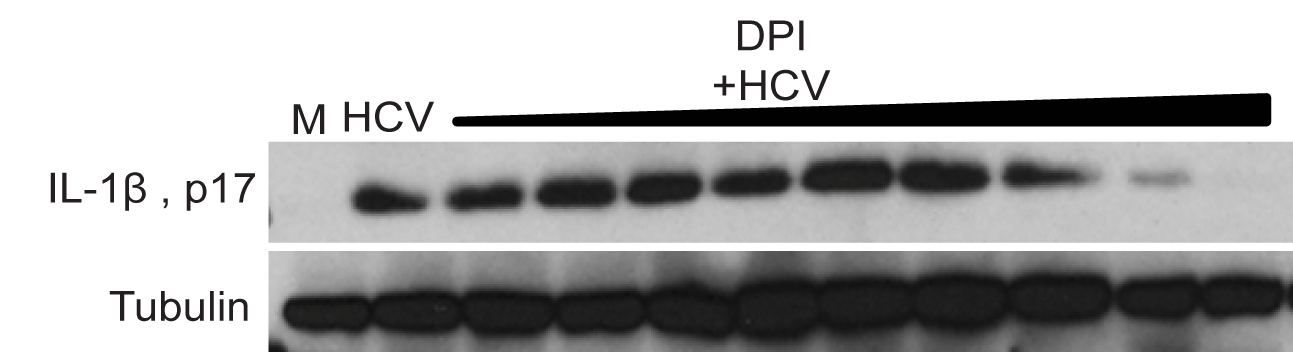

Supplement: Figure S10 — Differentiated THP-1 cells were, from left to right, mock treated, treated with HCV (moi = 0.01 based on Huh7 ffu) or pretreated with 0.3,0.6, 1.25, 2.5, 5, 10, 20, 40, 80 µM Diphenyleneiodonium chloride (DPI) for 1 hr followed by HCV treatment. 3 hr later cells were harvested and extracts subjected to immunoblot analysis for mature IL-1β and tubulin. (TIF) [file ppat.1003330.s010.tif]

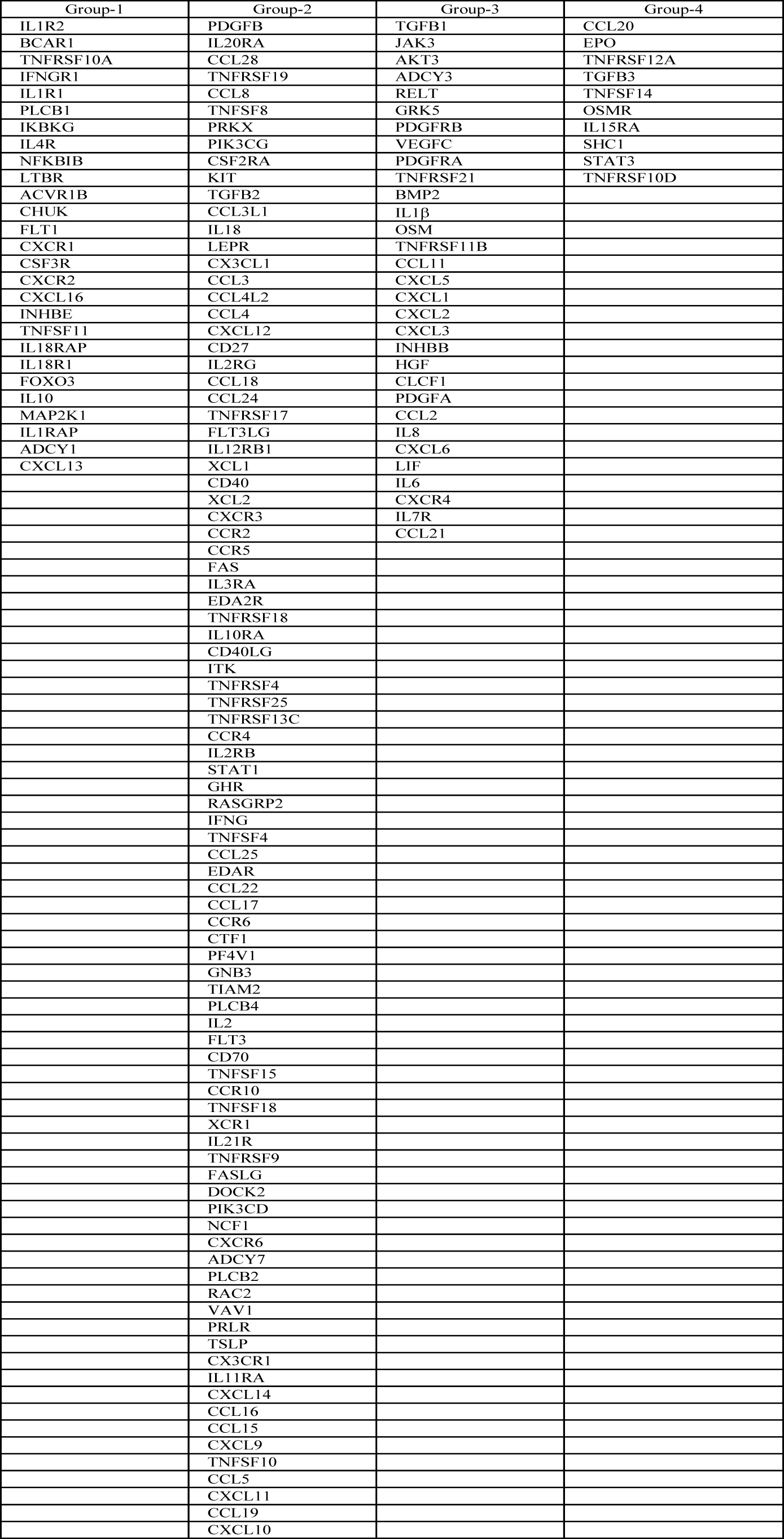

Supplement: Table S1 — Differentially expressed genes in liver biopsy specimens from chronic hepatitis C patients with mild (no fibrosis) and severe (cirrhosis) liver disease represented in ( Figure 1A ) Group-1 shows genes reduced in expression or “down-regulated” in association with HCV infection compared to control liver; group-2 shows genes with increased expression or “up-regulated” in HCV infection (mild and severe disease) compared to control liver; group-3 shows genes highly up-regulated in association with severe disease only compared to control liver; group-4 shows genes highly down-regulated in mild but not in severe disease patient livers. (TIF) [file ppat.1003330.s011.tif]

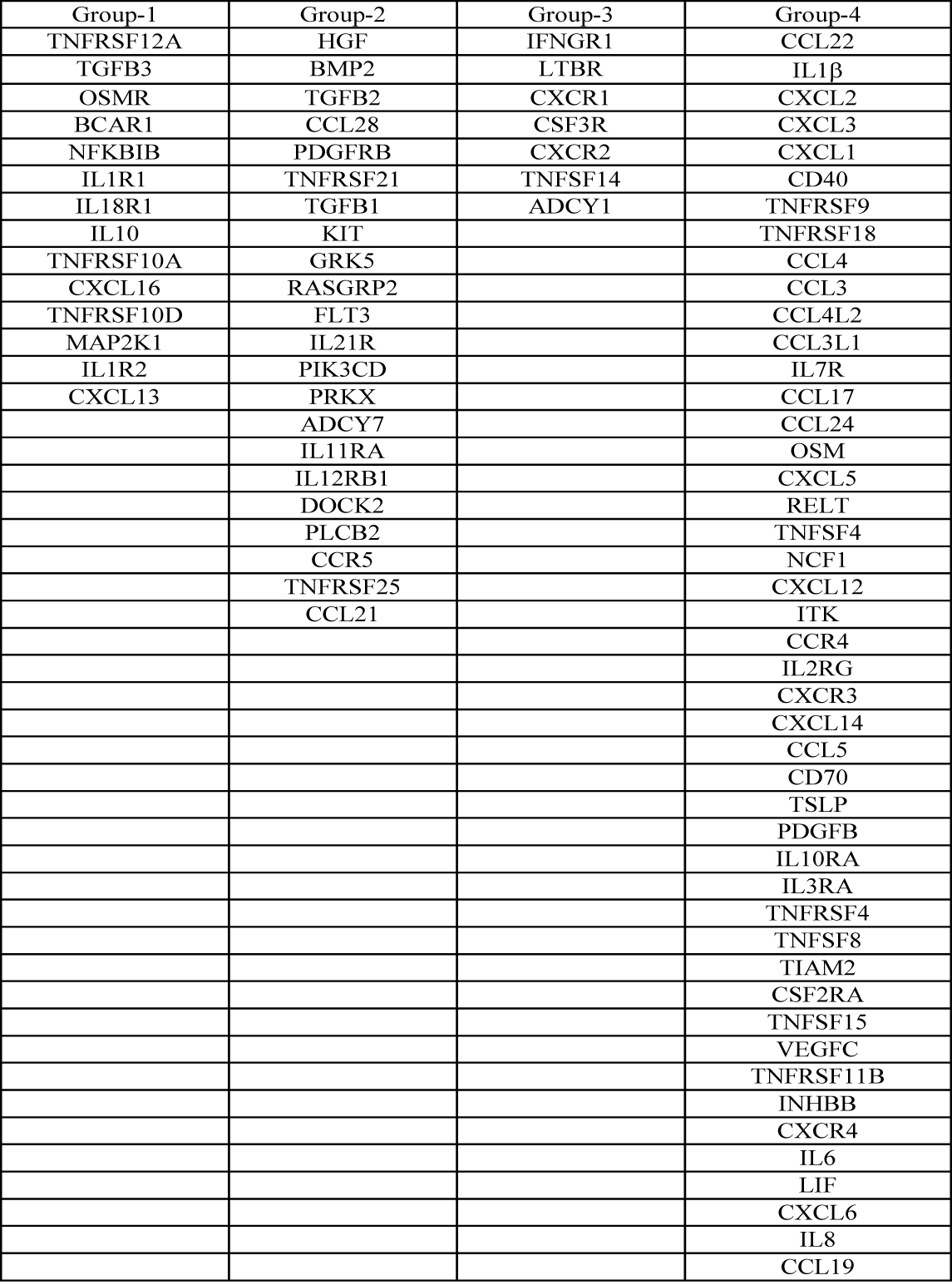

Supplement: Table S2 — Differentially expressed genes in chronic hepatitis C liver specimens with mild (no fibrosis) and severe (cirrhosis) disease or in THP-1 cells exposed to HCV represented in ( Figure 5B ). Group-1 shows genes up-regulated in HCV-exposed THP-1 cells only, as compared to mock-treated THP1 control cells; group-2 shows genes expressed in association with severe liver disease only but not in THP-1 cells; group-3 shows genes down-regulated in both hepatitis C liver and HCV-treated THP-1 cells; group-4 shows genes commonly expressed in both hepatitis C liver and HCV-treated THP-1 cells. Gene expression, as measured by RNA-seq analysis, in hepatitis C liver specimens was compared with control liver specimens. Gene expression in HCV-treated THP1 cells was compared with mock-treated THP1 cells. (TIF) [file ppat.1003330.s012.tif]
